# Mesenchymal stem cells with an enhanced antioxidant capacity integrate as smooth muscle cells in a model of diabetic detrusor underactivity

**DOI:** 10.1002/ctm2.70052

**Published:** 2024-10-10

**Authors:** Chae‐Min Ryu, YongHwan Kim, Jung‐Hyun Shin, Seungun Lee, Hyein Ju, Yun Ji Nam, Hyungu Kwon, Min‐Young Jo, Jinah Lee, Hyun Jun Im, Min Gi Jang, Ki‐Sung Hong, Hyung‐Min Chung, Sang Hoon Song, Myung‐Soo Choo, Seong Who Kim, Juhyun Park, Dong‐Myung Shin

**Affiliations:** ^1^ Center for Cell Therapy Asan Medical Center Seoul South Korea; ^2^ Department of Cell and Genetic Engineering, Asan Medical Center, Brain Korea 21 Project University of Ulsan College of Medicine Seoul South Korea; ^3^ Department of Urology, Mokdong Hospital Ewha Womans University Seoul South Korea; ^4^ Department of Stem Cell Biology, School of Medicine Konkuk University Seoul South Korea; ^5^ Mirae Cell Bio Co., Ltd. Seoul South Korea; ^6^ Department of Urology Asan Medical Center University of Ulsan College of Medicine Seoul South Korea; ^7^ Dr Joo Urology Clinic Seoul South Korea; ^8^ Department of Biochemistry and Molecular Biology, Asan Medical Center Brain Korea 21 Project, University of Ulsan College of Medicine Seoul South Korea

Dear Editor,

Diabetic cystopathy, particularly when it progresses to detrusor underactivity (DUA), poses significant clinical management challenges and affects a substantial number of individuals with diabetes mellitus (DM).^1^ Despite its prevalence, the etiology of diabetic DUA is poorly understood, and effective treatments are lacking. Our study addressed these gaps by investigating the mechanisms, tumorigenic risks, and optimal protocols of mesenchymal stem cell (MSC)^2^ transplantation in a preclinical model of diabetic DUA. Molecular signature of the transplanted cells in the pathological micro‐environments was characterised by single‐cell transcriptome analysis,^3^ emphasising the importance of the hepatocyte growth factor (HGF)–mesenchymal‐epithelial transition factor (MET) pathway and PD‐L1 in the mechanism for muscle regeneration and immunomodulation.

We reported the first clinical study of multipotent‐MSCs (M‐MSCs) derived from human embryonic stem cells (hESCs) for treating Hunner‐type interstitial cystitis, characterised by defective urothelium integrity and chronic inflammation.^4^ The hESC‐derived M‐MSCs were effective in a streptozotocin (STZ)‐induced diabetic DUA (STZ‐DUA) rat model.^5^ Transcriptomes of these preclinical samples were analysed to gain molecular insight into the pathogenesis of DM‐associated DUA and the mechanism of the MSC therapy (Figure 1A). Transcriptomes of STZ‐DUA bladders were distinct from those of sham‐operated bladders and also from those of STZ‐DUA rats administered M‐MSCs (Figure 1B), with 525 and 112 differentially expressed genes in the STZ‐DUA group relative to the sham and M‐MSC groups, respectively (Figure S1A–C).

**FIGURE 1:**
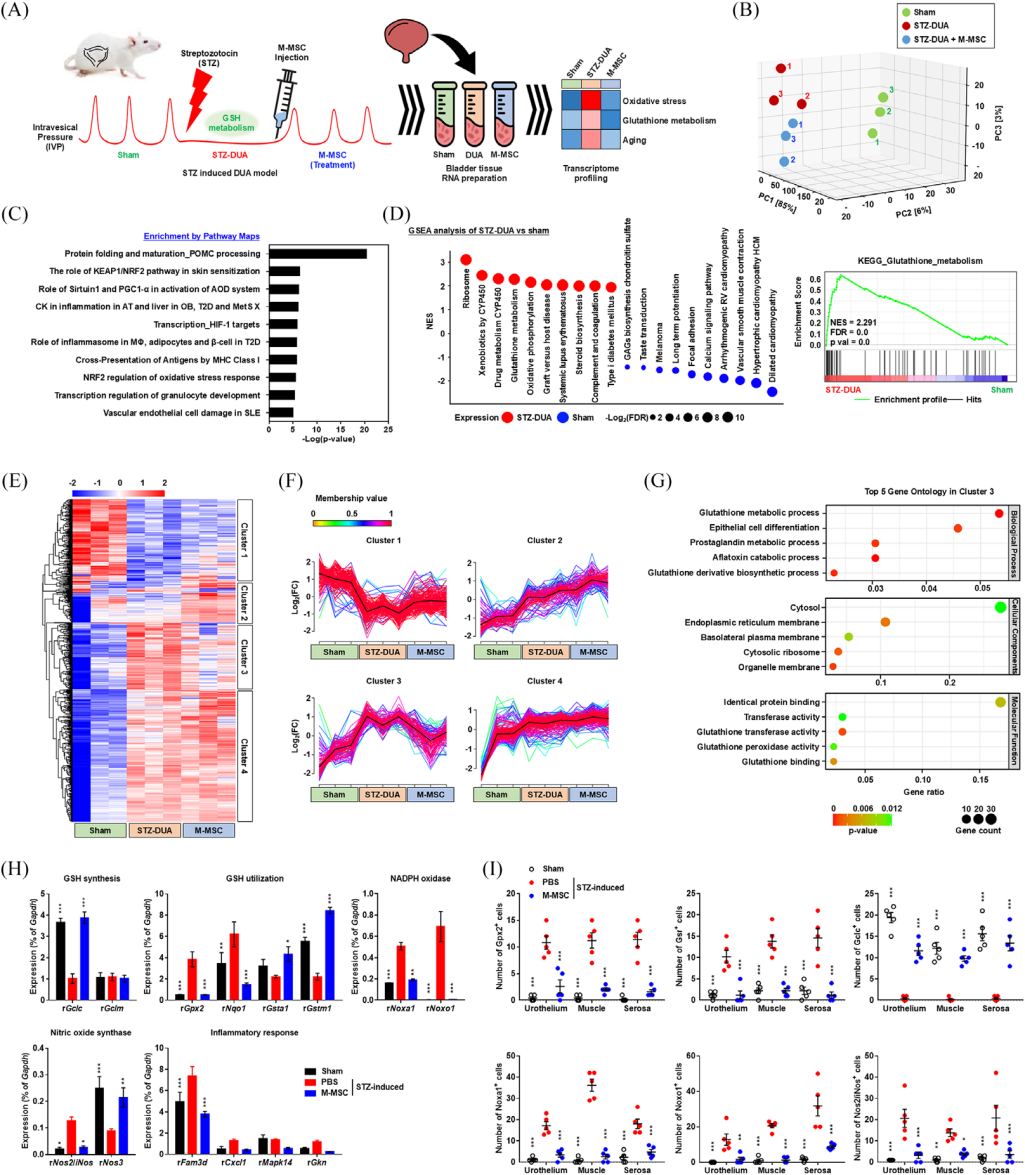
Transcriptome profiling of diabetic DUA following the MSC therapy. (A) Transcriptome analysis of bladder tissues from a preclinical study that demonstrated the effectiveness of M‐MSCs for treating diabetic DUA. (B) Principal component analysis (PCA) of the transcriptomes of the indicated groups. (C,D) The ten most highly enriched pathway maps identified by MetaCore analysis (C) and GSEA with a representative enrichment plot for glutathione (GSH) metabolism (D) in the comparison of transcriptomes between the STZ‐DUA and sham groups. (D) The bubble plot was presented by order of normalised enrichment score (NES) through size values by −Log2(FDR). As results of GSEA, the STZ‐DUA group was enriched with gene sets related to GSH metabolism (normalised enrichment score [NES] = 2.291) and drug metabolism by cytochrome p‐450 (CYP450) (NES = 2.327), as well as immunological disorders such as graft‐versus‐host disease (GVHD) (NES = 2.062) and systemic lupus erythematosus (NES = 2.051). (E) Heatmap of the differentially expressed genes (DEGs) generated using the pheatmap R package. (F) Mfuzz clustering analysis that identified four clusters of DEGs based on their patterns of expression. Mean expression values of each cluster are highlighted in black. The degree of membership of each gene for the cluster is presented as gradual membership values. (G) Gene ontology (GO) analysis (biological process, cellular component, and molecular function) of cluster‐3, which was upregulated in diabetic DUA, but their expression was normalised by M‐MSC therapy. In the bubble plot, the abscissa GeneRatio represents the proportion of enriched genes relative to the total number of genes. (H) qPCR analysis for the expression of genes involved in GSH synthesis, utilisation, NADPH oxidase, nitric oxide synthetase, or the inflammatory response. Expression is presented as the percentage relative to rat *Gapdh* expression (*n *= 5). (I) Quantitative analysis of immunofluorescence staining of GSH (upper panel) or oxidative stress (lower panel) related proteins in three locations (urothelium, muscle and serosa) of bladders from the indicated groups. Representative staining results for these proteins are available in Figure S4A–E. Statistical analyses were performed using a two‐way ANOVA with the Bonferroni post hoc test. ^*^ *p* < 0.05, ^**^ *p* < 0.01, ^***^ *p* < 0.001 relative to the STZ‐DUA group. The exact *p*‐values and number of replicates are specified in Data S1. DUA, Detrusor underactivity; MSC, mesenchymal stem cell; STZ, streptozotocin; GSEA, Gene‐set enrichment analysis.

Gene networks/pathways analysis by MetaCore indicated altered expression of genes involved in oxidative‐stress, inflammatory, and immune responses in the STZ‐DUA group (Figures 1C and S1D–F). Gene‐set enrichment analysis (GSEA) supported the significance of glutathione (GSH) metabolism and inflammatory responses in the pathogenesis of DM‐associated DUA, with gene‐sets related to muscle contraction and cardiomyopathy being downregulated in the STZ‐DUA group (Figure 1D and Table S1). Four gene clusters were observed in transcriptome changes following the M‐MSC therapy (Figure 1E,F). Cluster‐3 (131) genes were upregulated in diabetic DUA, but their expression was normalised by the M‐MSC therapy. The cluster‐3 genes predominantly involved in GSH‐related metabolic processes (Figures 1G and S1G–I).

For biomarkers from gene‐network (MetaCore) and leading‐edge (GSEA) analyses by comparing STZ‐DUA with M‐MSC groups, the M‐MSC therapy effectively coordinated the regulation of genes associated with GSH synthesis and metabolism (*Gclc* and *Gpx2*), activation of NADPH oxidase (*Noxa1*, *Noxo1*, and *Nox3*), nitric oxide synthesis (*Nos2*), and immune responses (Figure S2A–D and Table S2). The alternation in these biomarkers was validated by quantitative‐PCR (Figures 1H and S3) and immunofluorescence‐staining (Figures 1I and S4A–E) assays. Consistently, the levels of carbonylated proteins, a validated biomarker of oxidative‐stress were elevated in diabetic DUA (Figure S4F). The M‐MSC therapy alleviates these oxidative‐injuries in diabetic DUA. In vivo significance of these findings was validated by the beneficial outcomes of a GSH precursor/antioxidant N‐acetylcysteine^6^ alone and combination of the sub‐optimal dosage of M‐MSCs in the STZ‐DUA rat model (Figures 2A and S5–7). Collectively, these results provide in vivo proof of concept for the significance of oxidative‐injury in the pathogenesis of diabetic DUA and the mode of action of the MSC therapy.

**FIGURE 2:**
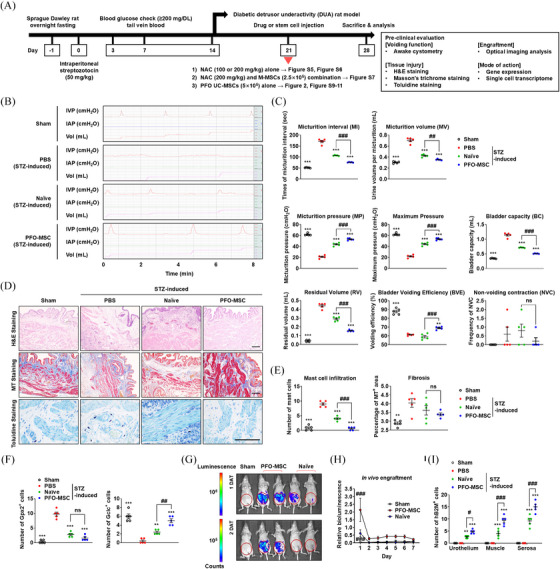
Improved in vivo therapeutic efficacy of PFO‐MSCs. (A) Schematic diagram of preclinical studies conducted in this study, which included (i) N‐acetylcysteine (NAC) alone, (ii) NAC combined with M‐MSCs, and (iii) PFO‐MSC therapies. The therapeutic outcomes of these treatments were evaluated using the awake cytometry, which allows long‐term evaluation of the bladder function in free‐moving animals. (B,C) Representative awake cystometry results (B) and quantitative voiding data (C) of sham‐operated and STZ‐DUA rats injected with the vehicle control (phosphate‐buffered saline, PBS) or naïve hUC‐MSCs or PFO‐MSCs (5.0 × 10^5^). Transplantation of naïve cultured hUC‐MSCs or PFO‐MSCs (5.0 × 10^5^) ameliorated the defective bladder functions in diabetic DUA, resulting in increases of micturition pressure (MP), maximum pressure, and bladder voiding efficiency (BVE) as well as decreases of micturition interval (MI), micturition volume (MV), bladder capacity (BC), and residual volume (RV). Importantly, compared with naïve cultured hUC‐MSCs, animals injected with PFO‐MSCs demonstrated significant enhancements of bladder function parameters, validating the improved therapeutic efficacy of these cells. (D) Hematoxylin and eosin staining (upper panel; magnification, 100×; scale bar, 200 µm), Masson's trichrome staining (middle panel; magnification, 100 ×; scale bar, 200 µm), and Toluidine blue staining (lower panel; magnification, 400×; scale bar, 100 µm) of bladder sections from the indicated groups at 1 week after injection of PFO‐MSCs. (E) Quantification of histological staining from five animals per group. (F) Quantitative analysis of immunofluorescence staining of Gpx2 and Gclc proteins in bladder sections of the indicated groups. (G,H) Representative images (G) and quantification (H) of bioluminescence activities of Nano‐lantern‐expressing UC‐MSCs (1.0 × 10^5^) subjected to normal (naïve) culture or the PFO procedure at the indicated day after transplantation (DAT) in the STZ‐induced diabetic mice model. (G) Representative images were obtained at 15 min after intraperitoneal injection of 150 µg/mL coelenterazine (200 µL), a substrate of Renilla luciferase. (H) Relative bioluminescence signals at the indicated DAT are presented as the relative values to 1 DAT value at the PFO‐MSC group. Nano‐lantern: a chimera of enhanced Renilla luciferase and Venus fluorescent protein with highly efficient bioluminescence resonance energy transfer. Notably, the animals injected with PFO‐MSCs showed significantly brighter bioluminescence throughout the entire experimental period than those injected with naïve hUC‐MSCs, indicating the in vivo engraftment capacity of PFO‐MSCs was superior. (I) Quantification results of immunostaining for hB2M throughout rat diabetic bladder sections from the indicated groups at 1 week after injection of PBS vehicle, naïve hUC‐MSCs, or PFO‐MSCs. All quantitative data are presented as the mean ± standard error of the mean (SEM) (*n* = 5). Data were analysed by the one‐way (C, E, F) or two‐way (H,I) ANOVA with the Bonferroni post hoc comparison (^**^ *p* < 0.01, ^***^ *p* < 0.001 relative to the PBS group; ^#^ *p* < 0.05, ^##^ *p* < 0.01, ^###^ *p* < 0.001). DUA, Detrusor underactivity; MSC, mesenchymal stem cell; STZ, streptozotocin; PFO, **P** rimed/**F** resh/**O** CT4; hUC, human umbilical‐cord.

Accordingly, we hypothesised that adult‐tissue derived MSCs with enhanced antioxidant capacity would ameliorate the pathological micro‐environment, have a high in vivo engraftment capacity, and show superior therapeutic efficacy.^7^, ^8^ Importantly, they are safer than hESC‐derivatives, which could provide advantages in clinical studies. Therefore, we investigated the benefits of human umbilical‐cord derived MSCs (hUC‐MSCs) using **P** rimed/**F** resh/**O** CT4 (PFO) procedure for treating diabetic DUA. As previously reported,^9^, ^10^ PFO‐MSCs, characterised by small size and high GSH dynamics, exhibited the reduced level of reactive oxygen species and cell death by oxidative‐stress (Figure S8).

Compared with naïve‐cultured hUC‐MSCs, animals injected with PFO‐MSCs demonstrated significant enhancements of bladder function parameters (Figure 2B,C), restoring histological injuries (Figures 2D,E, and S9A), and the alterations in expression of GSH‐related proteins in diabetic DUA (Figure 2F), validating their improved therapeutic efficacy. All these beneficial effects were sustained for 2 or 4 weeks after a single transplantation of PFO‐MSCs (Figure S10), proving their long‐lasting therapeutic effects on diabetic DUA. Longitudinal µ‐PET/MRI bio‐imaging analysis over a 9‐month period revealed little tumorigenic potential of PFO‐MSCs following injection (Figure S9B).

PFO‐MSCs exhibited superior in vivo engraftment capacity and retention kinetics (Figures 2G,H and S11A). These findings were confirmed by immunofluorescence‐staining of human  β2‐microglobin (hB2M), with more hB2M^+^ cells detected after PFO‐MSC transplantation (Figure 2I and S11B). The engrafted hB2M^+^ PFO‐MSCs integrated as NG2 expressing pericytes around muscle fibres (Figures S11C and S12A) and directly differentiating into myocytes (Figure 3A), actively participating in muscle repair. The hB2M^+^/α‐SMA^+^ cells persisted for 2 or 4 weeks following PFO‐MSC transplantation (Figure S12B,C).

**FIGURE 3:**
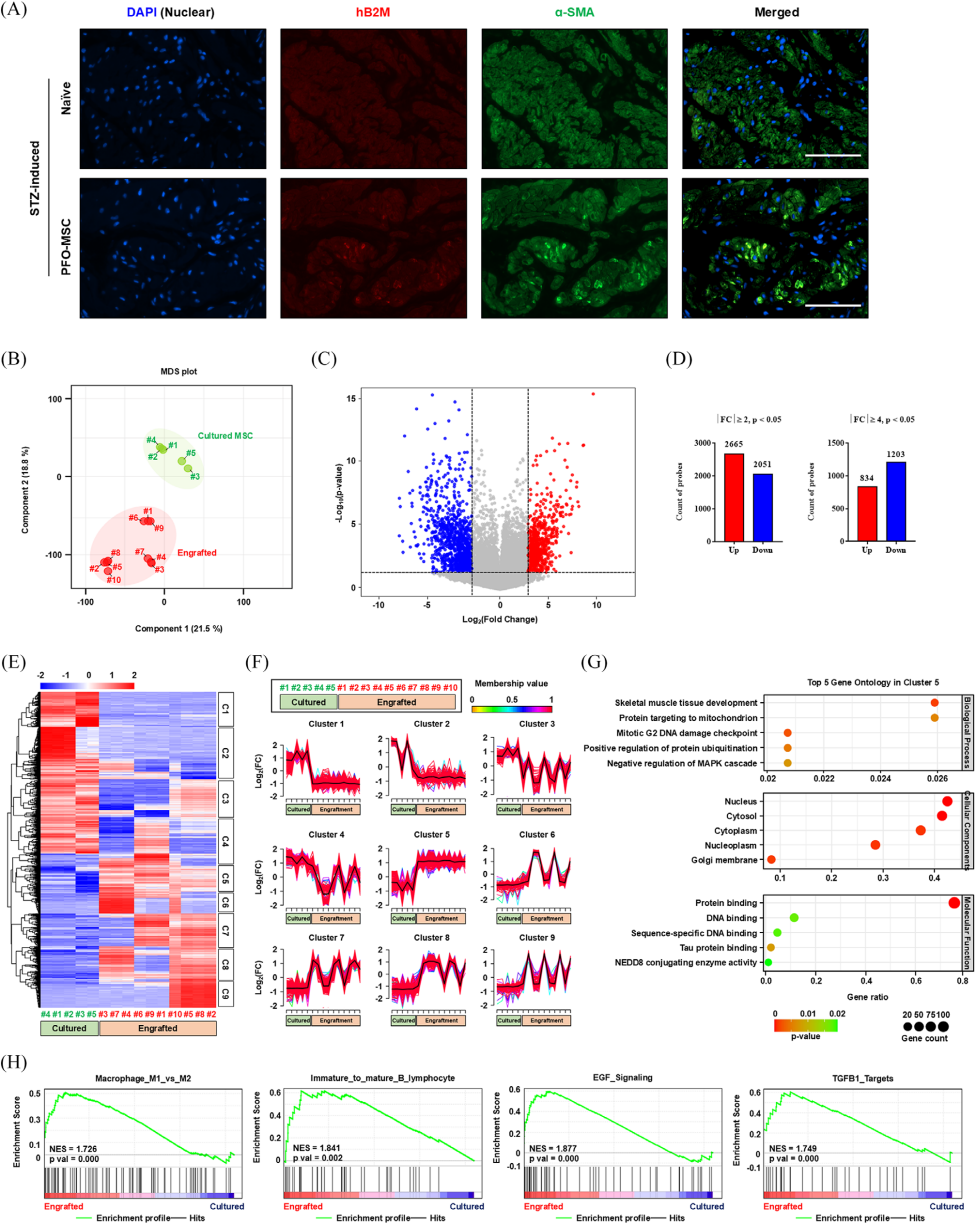
Single‐cell transcriptome profiling of engrafted PFO‐MSCs. (A) Representative immunofluorescence (magnification, 400×; scale bar, 200 µm) micrographs of co‐staining of hB2M (red) and α‐smooth muscle actin (α‐SMA, blue), a muscle marker in the indicated bladder sections of STZ‐DUA rats at 1 week after transplantation. Nuclei were counterstained with 4′,6‐diamidino‐2‐phenylindole (DAPI) (blue). (B,C) PCA (B) and volcano plot (C) comparing five cultured (Cultured_#1–5) and ten engrafted (Engrafted_#1–10) single‐cell transcriptomes. (D) The number of upregulated and downregulated genes with the indicated cut‐off values of |log2(FC)| > 2 or 4 and *p* < 0.05. Accordingly, ∼2000 DEGs, including 834 upregulated and 1203 downregulated DEGs, were identified in engrafted cells compared with cultured cells. (E) Heatmap of DEGs in single‐cell transcriptomes generated using the pheatmap R package with nine gene clusters. Cluster‐1 was downregulated and cluster‐5 was upregulated in engrafted cells compared with cultured cells. (F) Mfuzz clustering analysis that identified nine clusters of DEGs based on their patterns of expression. Mean expression values of each cluster are highlighted in black. (G) GO analysis (biological process, cellular component, and molecular function) of cluster‐5, which was upregulated in engrafted cells compared with cultured cells. In the bubble plot, the abscissa GeneRatio represents the proportion of enriched genes to the total number of genes. Notably, GO analysis of cluster‐5 genes indicated that the single‐cell transcriptomes of engrafted cells were characteristically represented by pathways related to skeletal muscle tissue development and cellular stress such as the DNA damage checkpoint and mitogen‐activated protein kinase (MAPK) cascade. (H) GSEA with a representative enrichment plot for the immune response and growth factors in the comparison of single‐cell transcriptomes between the cultured and engrafted groups. Accordingly, engrafted cells were enriched with gene sets related to the immune response including macrophage subtypes (NES = 1.726) and B lymphocyte maturation (NES = 1.841), as well as growth factors including epidermal growth factor (EGF) (NES = 1.877) and transforming growth factor beta (TGFB) signalling (NES = 1.749), compared with cultured cells. DUA, Detrusor underactivity; MSC, mesenchymal stem cell; STZ, streptozotocin; PFO, **P** rimed/**F** resh/**O** CT4; hUC, human umbilical‐cord; PCA, principal component analysis; DEG, differentially expressed gene; GSEA, Gene‐set enrichment analysis; NES, normalised enrichment score.

Single‐cell transcriptome profiling revealed the molecular characteristics of engrafted PFO‐MSCs within the pathological micro‐environment (Figure S13A,B). The engrafted PFO‐MSCs had distinct molecular characteristics from cultured cells (Figure 3B–D), with alterations in genes related to muscle progenitor cells, HGF‐signalling, cell‐adhesion, and immune responses (Figure S13C), with distinct nine gene clusters (Figure 3E,F). Cluster‐1 was downregulated in engrafted cells by enriching apoptosis and cell division genes (Figure S13D,E). Cluster‐5, up‐regulated in engrafted cells, showed an enrichment of genes associated with skeletal muscle development and immunomodulation (Figures 3E–H and S13F), elucidating the mode of action of the PFO‐MSC therapy.

Gene‐network and leading‐edge biomarker analyses identified HGF–MET and PD‐L1 as key genes representing muscle regeneration and immunomodulatory processes, respectively (Figure 4A,B, S14, Table S3, and S4). In immunostaining results, the hB2M^+^/MET^+^ cells were mainly located within muscle bundles (Figures 4C and S15A) and hB2M^+^ cells, situated within or nearby muscle bundles, robustly expressed RHOA (Figures 4D and S15B) and PAX7 or MYOD1 muscle markers (Figures S15C,D), indicating the direct roles of the engrafted cells in muscle regeneration. Furthermore, hB2M^+^/PD‐L1^+^ engrafted cells were found in various bladder locations, including the stroma around blood vessels and within muscle bundles, supporting their immunosuppressive capacity (Figure 4E,F).

**FIGURE 4:**
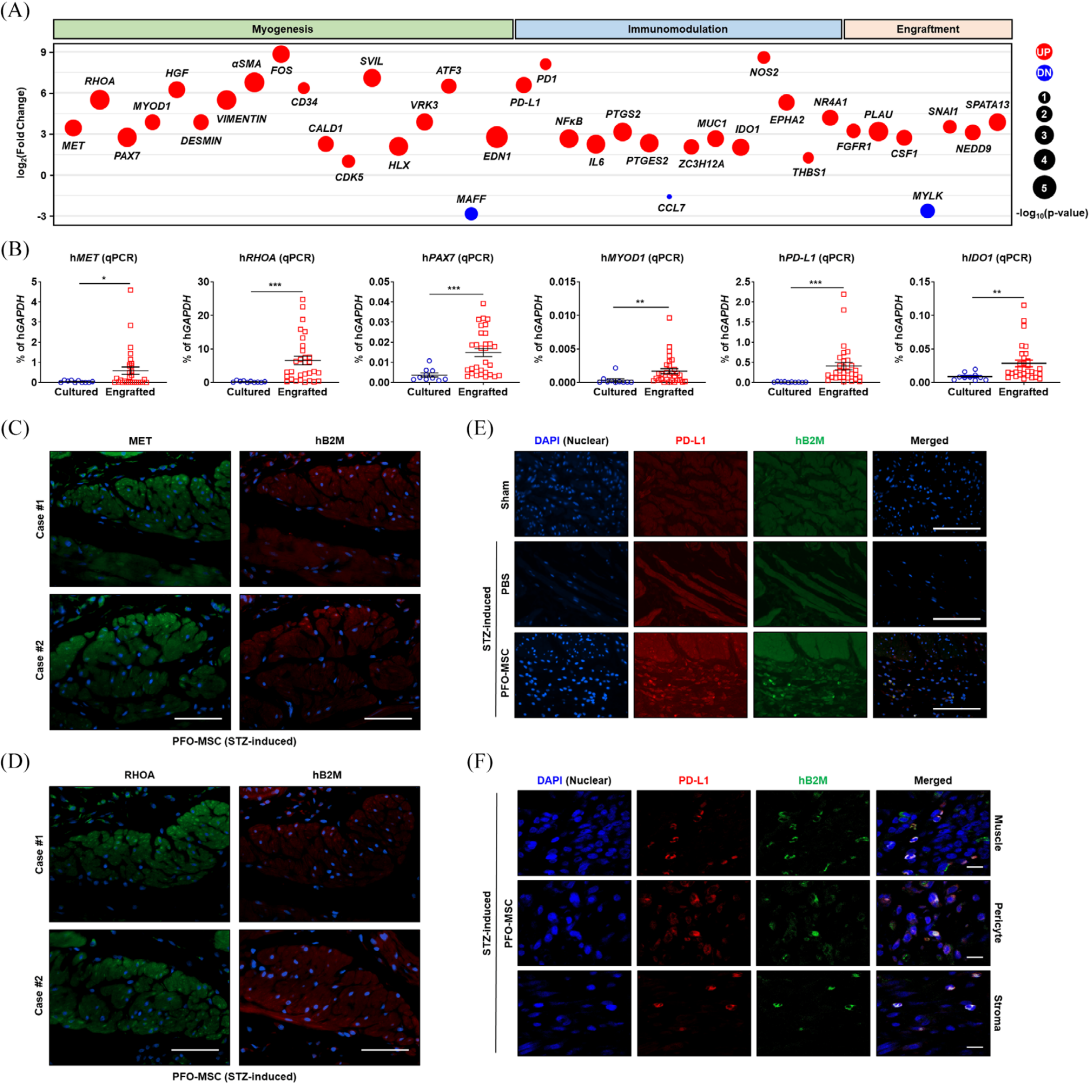
Upregulation of MET and PD‐L1 in engrafted M‐MSCs. (A) Bubble plot of qPCR results for putative biomarkers characterising engrafted PFO‐MSCs in diabetic DUA, which are related to myogenesis, immunomodulation, and engraftment processes. (B) qPCR analysis validating expression of biomarker genes representing each process (Cultured *n *= 10, Engrafted *n *= 30). Expression is presented as the percentage relative to human *GAPDH* expression. All quantitative data are shown as the mean ± SEM. Statistical significance was examined by the non‐parametric Mann–Whitney test (^*^ *p* < 0.05, ^**^ *p* < 0.01, ^***^ *p* < 0.001). (C,D) Immunostaining of MET (C) or RHOA (D) (green) as well as hB2M (red) in bladder sections of an STZ‐DUA rat at 1 week after injection of PFO‐MSCs. Two independent images are shown (magnification, 400×; scale bar, 200 µm). (E,F) Representative fluorescence (magnification, 400×; scale bar, 200 µm, E) and confocal (magnification, 1000×; scale bar, 10 µm, F) micrographs of co‐staining of PD‐L1, an immunomodulation marker (red), and hB2M (green) in bladder sections of the indicated groups. Nuclei were stained with DAPI (blue). DUA, Detrusor underactivity; MSC, mesenchymal stem cell; STZ, streptozotocin; PFO, **P** rimed/**F** resh/**O** CT4.

In summary, comprehensive understanding of the key pathological mechanisms of diabetic DUA can guide the selection of optimal stem cells for therapeutic efficacy and safety. Our study demonstrates that hUC‐derived PFO‐MSCs, with enhanced GSH dynamics and engraftment capacity, effectively alleviate tissue‐injury and contribute to muscle regeneration and immunomodulation in diabetic DUA, providing a promising approach for clinical translation of stem cell therapies (Figure S16). The significance and limitations of this study are discussed in detail in the Supporting information.

## AUTHOR CONTRIBUTIONS

Chae‐Min Ryu, YongHwan Kim, and Jung‐Hyun Shin contributed equally to this work. *Conceptualisation*: Dong‐Myung Shin, S.H.K., Juhyun Park, and Myung‐Soo Choo. *Methodology*: Dong‐Myung Shin, Chae‐Min Ryu, Yong Hwan Kim, and Jung‐Hyun Shin. *Investigation*: Chae‐Min Ryu, Yong Hwan Kim, Jung‐Hyun Shin, Seungun Lee, Hyein Ju, Yun Ji Nam, Hyungu Kwon, Min‐Young Jo, Jinah Lee, Hyun Jun Im, and Min Gi Jang. *Writing—original draft*: Dong‐Myung Shin, Chae‐Min Ryu, Yong Hwan Kim, and Jung‐Hyun Shin. *Writing—review & editing*: Dong‐Myung Shin, Juhyun Park, Chae‐Min Ryu, Yong Hwan Kim, Jung‐Hyun Shin, Seong Who Kim, and Sang Hoon Song. *Funding Acquisition*: Dong‐Myung Shin, Chae‐Min Ryu, Jung‐Hyun Shin, and Seong Who Kim. *Resources*: Ki‐Sung Hong and Hyung‐Min Chung. *Data curation*: Dong‐Myung Shin, Chae‐Min Ryu, Yong Hwan Kim, Seong Who Kim, and Jung‐Hyun Shin. *Supervision*: Dong‐Myung Shin, Juhyun Park, and Seong Who Kim.

## CONFLICT OF INTEREST STATEMENT

D‐.M.S. cofounded Cell2in, a company focused on developing FreSHtracer‐based GRC assays. The other authors declare that no conflicts of interest exist.

## ETHICS STATEMENT

All activities were conducted in compliance with the guidelines of the Ethics Committee on the Use of Human Subjects at Asan Medical Center (IRB#: 2015‐0303). Approval for animal experiments was granted by the Institutional Animal Care and Use Committee of the University of Ulsan College of Medicine (IACUC‐2020‐12‐160). All procedures adhered to the applicable regulations and guidelines.

## Supporting information



Supporting information

Supporting information
