# Supplementary material for: Mesenchymal stem cells with an enhanced antioxidant capacity integrate as smooth muscle cells in a model of diabetic detrusor underactivity
Source: Clin Transl Med. 2024 Oct 10;14(10):e70052. doi: 10.1002/ctm2.70052 (PMC11467036; doi:10.1002/ctm2.70052)
Supplement: Supplementary file 2 — Supporting information [file CTM2-14-e70052-s001.pdf]

Supplementary Information

Mesenchymal stem cells with an enhanced antioxidant capacity integrate as smooth muscle cells in a model of diabetic detrusor underactivity

Corresponding authors. d0shin03@amc.seoul.kr, urojpark@amc.seoul.kr, and swhokim@amc.seoul.kr

This PDF file includes:

Figures S1 to S16 and figure legends

Table S1 to S5 and table legends

Key resource table

Supplementary Methods

Supplementary Notes

Supplementary References

Other Supplementary Information include a separate file including

Table S1–5

Dataset S1. Source data for quantification analyses

SUPPLEMENTARY FIGURE LEGENDS

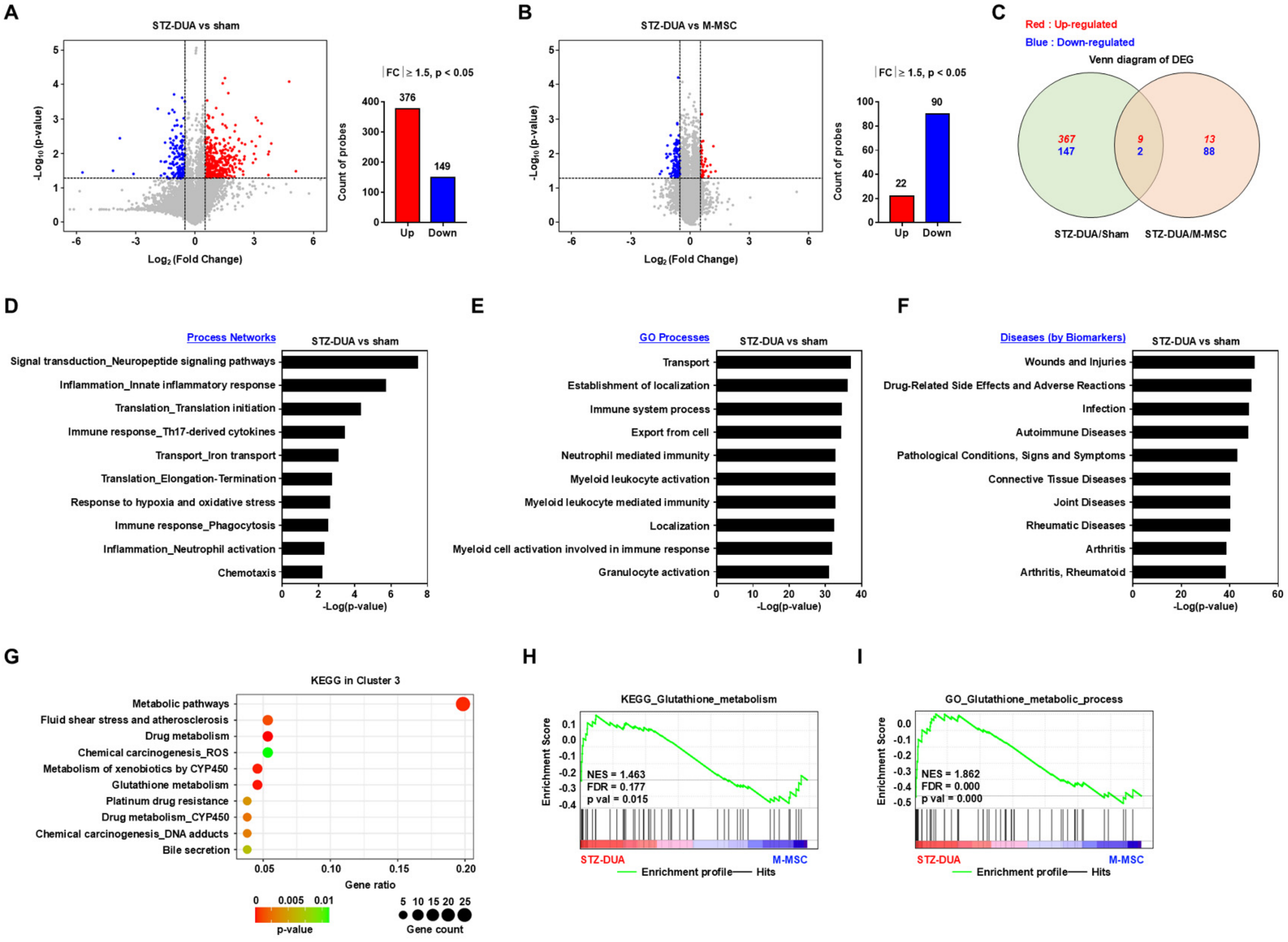

Figure S1. Transcriptome features of diabetic DUA following MSC therapy

(A–C) Volcano plots **(A and B)** and Venn diagram **(C)** comparing the transcriptome of the STZ-DUA group with that of the sham **(A)** or M-MSC **(B)** group. **(A and B)** The number of upregulated and downregulated genes with the indicated cut-off values is shown in the right panel. Accordingly, 525 differentially expressed genes (DEGs), including 376 upregulated and 149 downregulated DEGS, were identified in the STZ-DUA group relative to the sham group

with the criteria of │fold change│ > 1.5 and p<0.05. There were 112 DEGs, including 22 upregulated and 90 downregulated genes, in the comparison of the STZ-DUA and M-MSC groups. DEGs did not markedly overlap between the two comparisons, with only nine upregulated and two downregulated genes commonly identified. **(D–F)** The ten most highly enriched process networks **(D)**, GO processes **(E)**, and disease biomarkers **(F)** in MetaCore analysis comparing the transcriptomes of the STZ-DUA and sham groups. Compared with the sham group, bladders in the STZ-DUA group were characterized by altered expression of genes involved in NRF-mediated oxidative stress responses as well as biological processes related to inflammatory and immune responses. **(G)** KEGG enrichment analysis of cluster-3. In the bubble plot, the abscissa GeneRatio represents the proportion of enriched genes relative to the total number of genes. Accordingly, GSH-related metabolic pathways and drug/xenobiotic metabolism via CYP450-related pathways were highly ranked among cluster-3 genes **(H and I)** Representative enrichment plots characterizing GSH metabolism in KEGG **(I)** and GO **(J)** analyses in the comparison of transcriptomes between the STZ-DUA and M-MSC groups. Notably, M-MSC therapy effectively reinstated GSH-related metabolism and oxidative stress pathways that were significantly perturbed in diabetic DUA compared with the sham group. Detailed information about the MetaCore and GSEA analysis results is presented in **Dataset 1**.

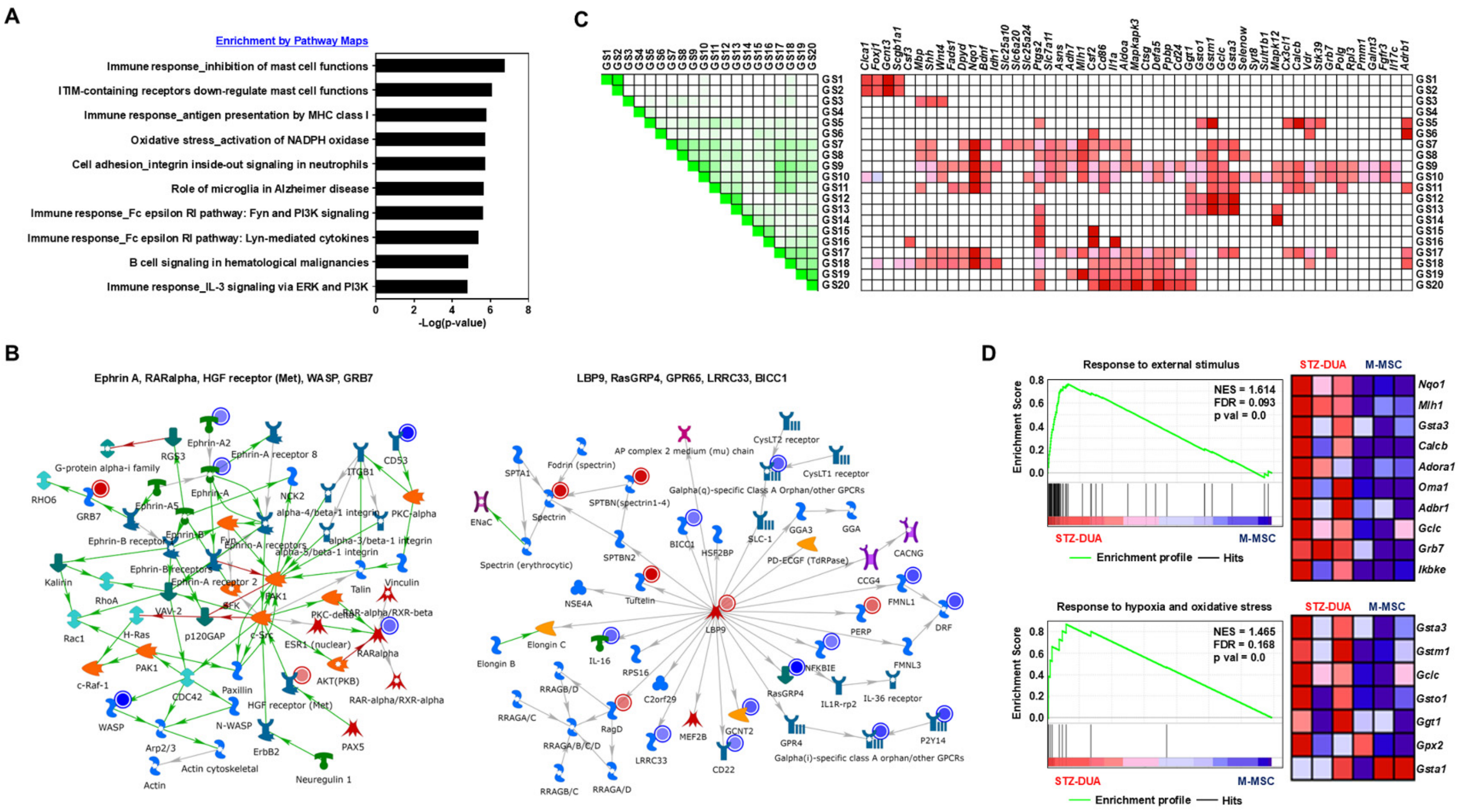

Figure S2. Biomarkers characterizing the mechanism by which MSC therapy improves diabetic DUA

(A and B) The top ten pathway maps **(A)** and two representative HGF (left panel)- and RasGRP4 (right panel)-associated gene networks determined via MetaCore analysis comparing the STZ-DUA and M-MSC groups. Gene networks are illustrated by overlaying experimental values as fold changes. Upregulated and downregulated genes are indicated in red and blue, respectively. **(C)** GSEA leading-edge analysis of transcriptomes using the top 20 gene sets enriched in the comparison of transcriptomes between the STZ-DUA and M-MSC groups. Expression of several GSH-related genes significantly differed between the STZ-DUA and M-MSC groups, as evidenced by clustering of the GSH-mediated redox homeostasis gene sets. **(D)** Representative enrichment plots for GSEA comparing the transcriptomes of the STZ-DUA

and M-MSC groups. Heatmaps containing the top ten genes in each enrichment plot are depicted in the right panel, with upregulated and downregulated genes indicated in red and blue, respectively. GSEA, gene set enrichment analysis; ES, enrichment score; NES, normalized ES; NOM p-val, nominal p-value; FDR, false discovery rate. Detailed information about the MetaCore and GSEA results is presented in **Dataset 1**.

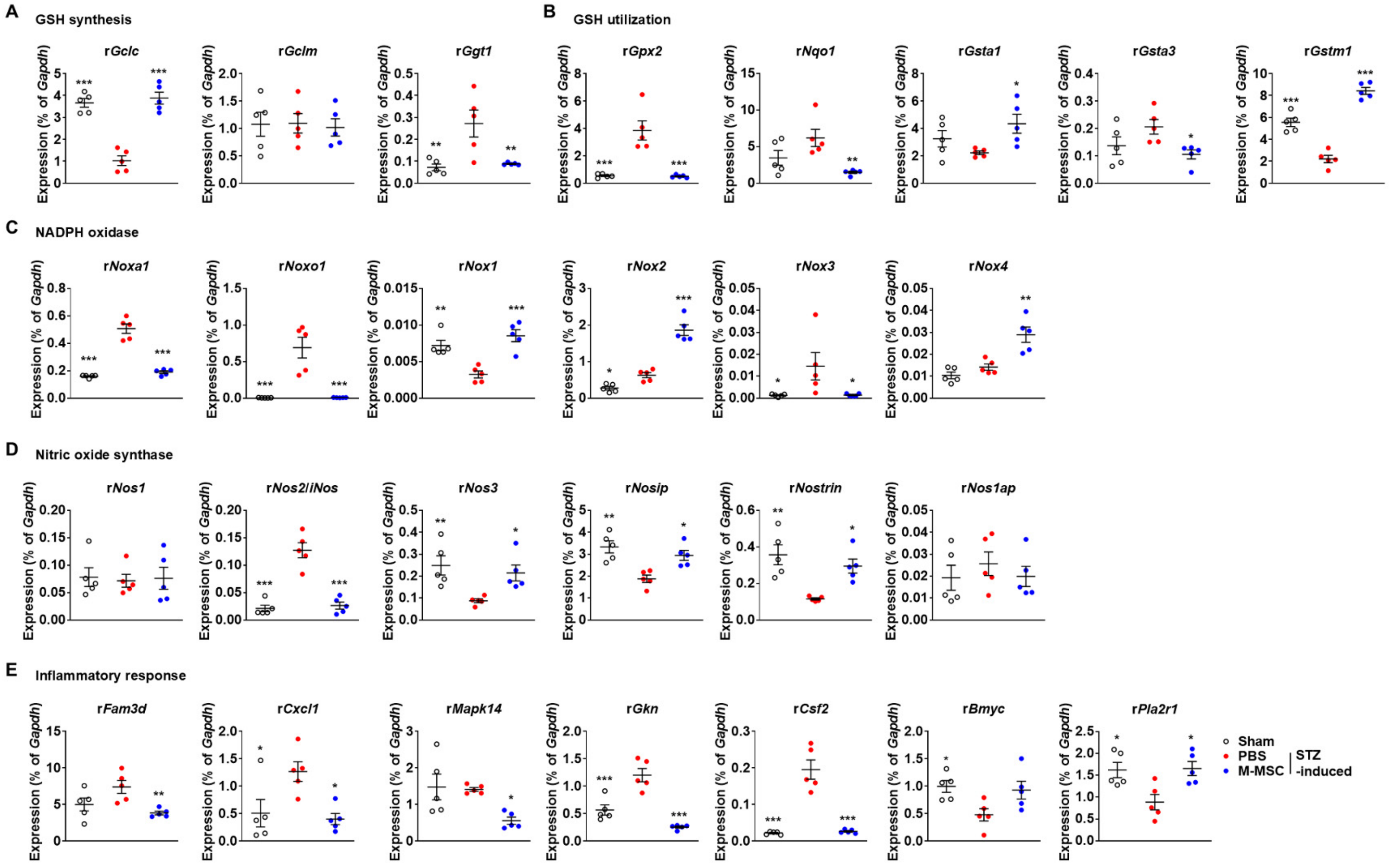

Figure S3. Gene expression analysis of the diabetic DUA following MSC therapy.

(A–E) qPCR analysis of genes related to GSH synthesis **(A)**, GSH utilization **(B)**, NADPH oxidase **(C)**, nitric oxide synthetase **(D)**, and inflammatory response **(E)**. Expression is presented as the percentage relative to rat *Gapdh* expression and shown as a dot plot of the mean ± SEM (n = 5). Statistical significance was examined by the Bonferroni post-hoc test (*p<0.05, **p<0.01, ***p<0.001 compared with the STZ-DUA group).

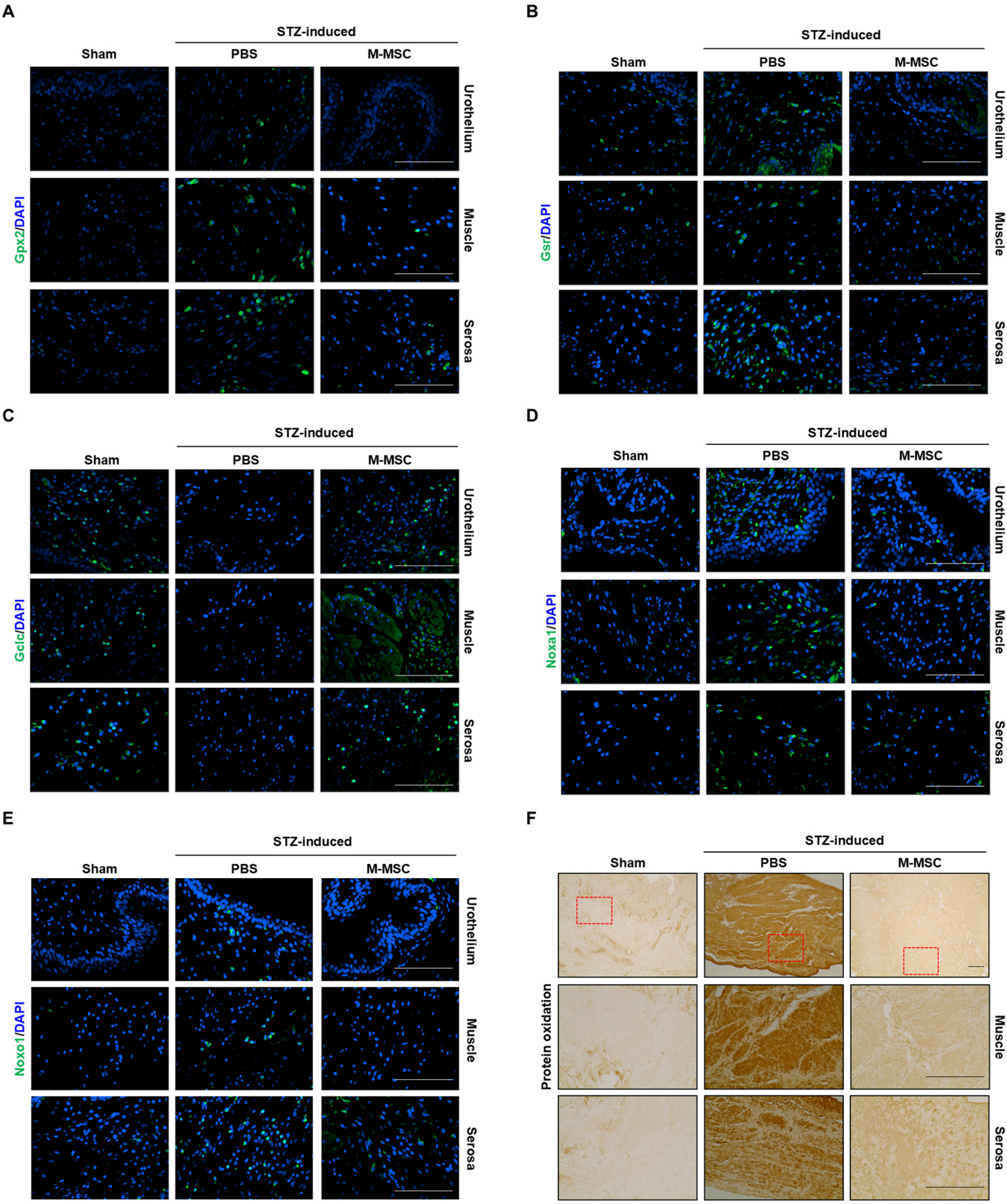
A
STZ-induced
Sham
PBS
M-MSC
Urothelium
Muscle
Serosa
Gpx2/DAPI
B
STZ-induced
Sham
PBS
M-MSC
Urothelium
Muscle
Serosa
Gsr/DAPI
C
STZ-induced
Sham
PBS
M-MSC
Urothelium
Muscle
Serosa
Gclc/DAPI
D
STZ-induced
Sham
PBS
M-MSC
Urothelium
Muscle
Serosa
Noxa1/DAPI
E
STZ-induced
Sham
PBS
M-MSC
Urothelium
Muscle
Serosa
Noxo1/DAPI
F
STZ-induced
Sham
PBS
M-MSC
Muscle
Serosa
Protein oxidation

Figure S4. Beneficial effects of M-MSC therapy on the histological characteristics of oxidative injury in diabetic DUA

(A–E) Representative images of immunofluorescence staining of GSH metabolism proteins [Gpx2 **(A)**, Gsr **(B)**, and Gclc **(C)**] and NAPDH oxidases [Noxa1 **(D)** and Noxo 1 **(E)**] throughout three locations (urothelium, muscle, and serosa) in bladder sections of the indicated groups (magnification, 400×; scale bar, 200 μm). Nuclei were counterstained with DAPI (blue). Quantification data for these immunostaining results are available in **Figure 1I**. **(F)** Representative images of histological examination of proteins with carbonyl groups incorporated by oxidative damage in bladder tissues (magnification, 40× (upper panel) and 400× (lower panel); scale bar, 200 μm). Notably, M-MSC therapy ameliorated the alterations in these GSH-related proteins, NADPH oxidases, and the elevated levels of carbonylated proteins, a validated biomarker of oxidative stress, in diabetic DUA, indicating that M-MSC therapy alleviates oxidative injury in diabetic DUA.

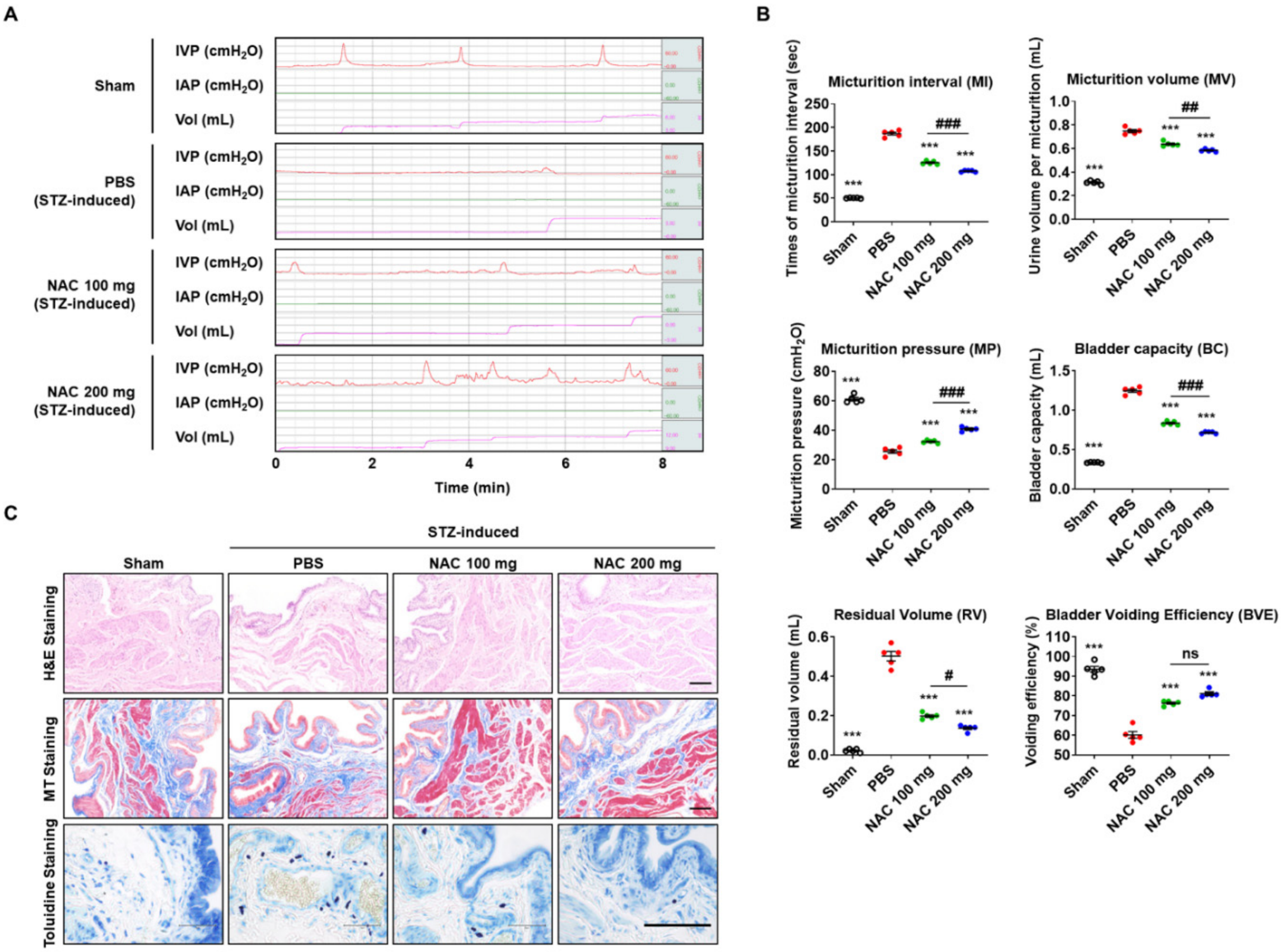

Figure S5. Therapeutic efficacy of NAC monotherapy for diabetic DUA

(A and B) Representative awake cystometry results **(A)** and quantitative bladder voiding parameters **(B)** at 1 week after injection of NAC (100 or 200 mg/kg). Notably, treatment with NAC dose-dependently improved the voiding dysfunction in diabetic DUA, including increases in the micturition interval (MI), micturition volume (MV), bladder capacity (BC), and residual volume (RV), as well as decreases in micturition pressure (MP) and bladder voiding efficiency (BVE). IVP, intravesical pressure; IAP, intra-abdominal pressure. **(C and**

D) Histological analysis of the effects of NAC administration on STZ-induced DUA injury, including i) H&E staining (magnification, 100×; scale bar, 100 μm), ii) Masson's trichrome staining (magnification, 100×; scale bar, 100 μm), and iii) Toluidine blue staining (magnification, 400×; scale bar, 200 μm) in the indicated bladder tissues. Nuclei were stained with Mayer's hematoxylin. In line with the results of awake cystometry, the beneficial effects of NAC treatment were validated by the histological examination, which showed that several histological changes observed in STZ-DUA rats, including loss of the muscular layer, tissue fibrosis, and mast cell infiltration, were prevented. All quantitative data are presented as the mean ± SEM (*n* = 5). Data were analyzed using a one-way ANOVA with the Bonferroni post-hoc comparison (***p<0.001 relative to the PBS vehicle group; #p<0.05, ##p<0.01, ###p<0.001, ns, non-significant). The exact p-values and number of replicates are specified in **Data S1**.

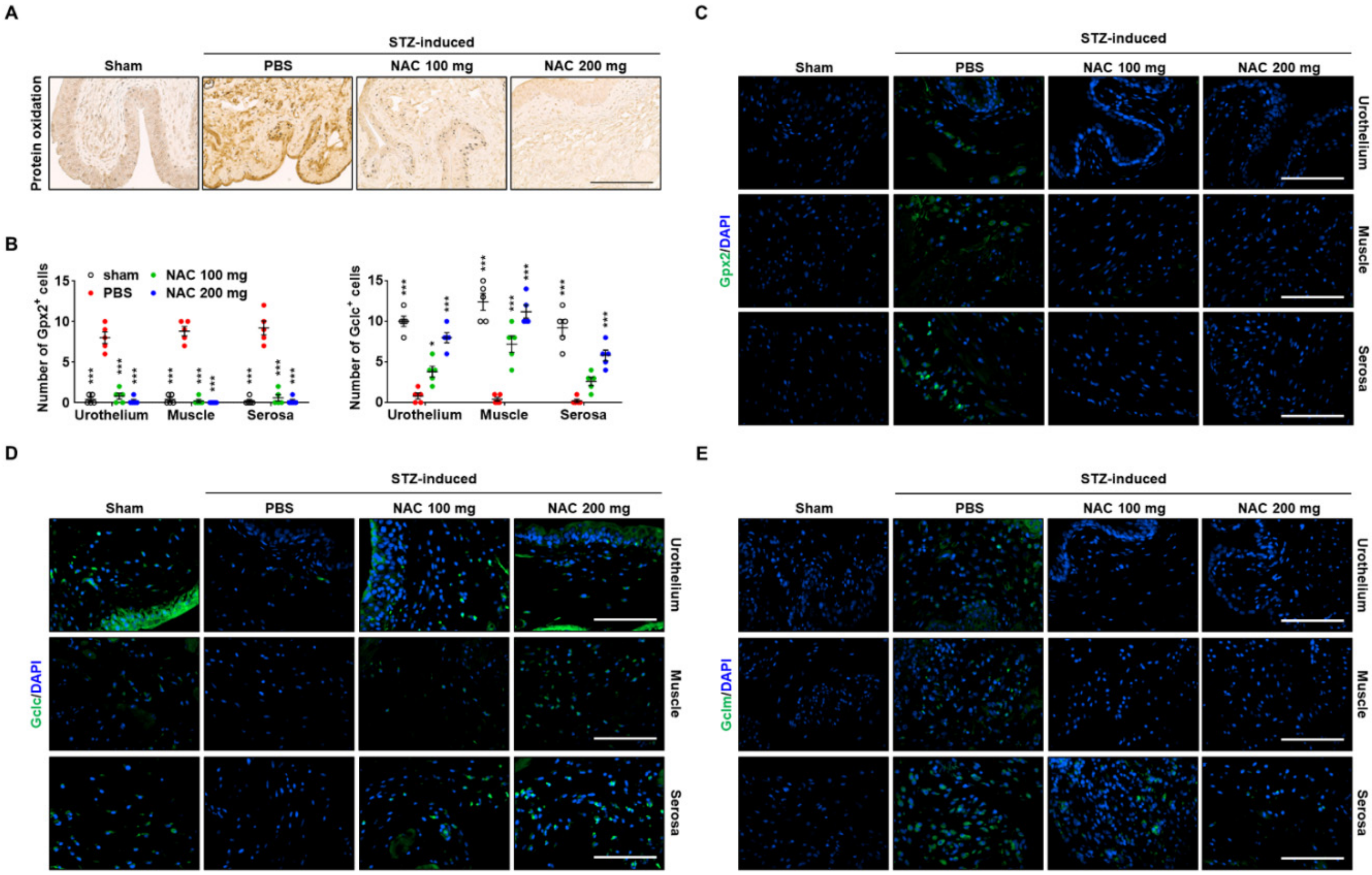

Figure S6. Histological analysis of NAC monotherapy

(A) Histological analysis of Oxyblot staining (magnification, 400×; scale bar, 200 μm) to detect oxidative damaged proteins in the indicated bladder tissues. **(B–E)** Quantitative analysis **(B)** and representative images **(C–E)** of immunofluorescence staining for Gpx2 (C), Gclc (D), and Gclm **(E)** proteins throughout three locations (urothelium, muscle, and serosa) of bladder sections. Similar to M-MSC therapy, NAC treatment significantly inhibited accumulation of carbonylated proteins and alterations in expression of GSH-related proteins in diabetic DUA. Quantitative data are presented as the mean ± SEM (n = 5). Data were analyzed using a two-way ANOVA with the Bonferroni post-hoc comparison (*p<0.05, **p<0.01, ***p<0.001 relative to the PBS vehicle group; #p<0.05, ##p<0.01, ###p<0.001). The exact p-values and

number of replicates are specified in **Data S1**.

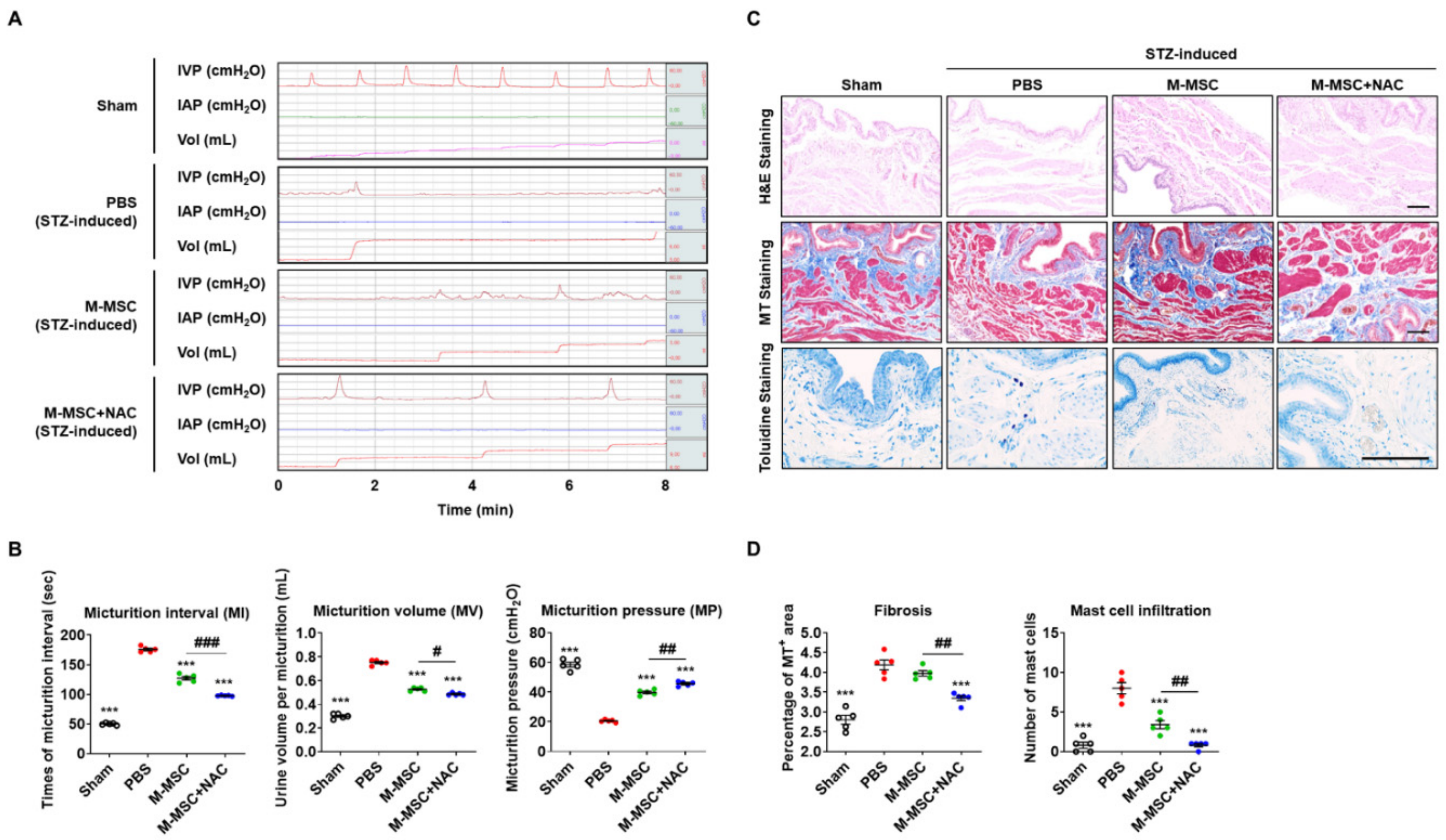

Figure S7. Therapeutic efficacy of combination therapy with NAC and M-MSCs

(A and B) Representative awake cystometry results **(A)** and quantitative bladder voiding parameters **(B)** at 1 week after injection of M-MSCs (2.5×10^5) alone or in combination with NAC (200 mg/kg). **(C)** Histological analysis including i) H&E staining (magnification, 100×; scale bar, 100 μm), ii) Masson's trichrome staining (magnification, 100×; scale bar, 100 μm), and iii) Toluidine blue staining (magnification, 400×; scale bar, 200 μm) in the indicated bladder tissues. Nuclei were stained with Mayer's hematoxylin. **(D)** Quantification of histological staining from five animals per group. Quantitative data are presented as the mean ± SEM (n = 5). Data were analyzed using a one-way ANOVA with the Bonferroni post-hoc comparison ***p<0.001 relative to the PBS vehicle group; #p<0.05, ##p<0.01, ###p<0.001).

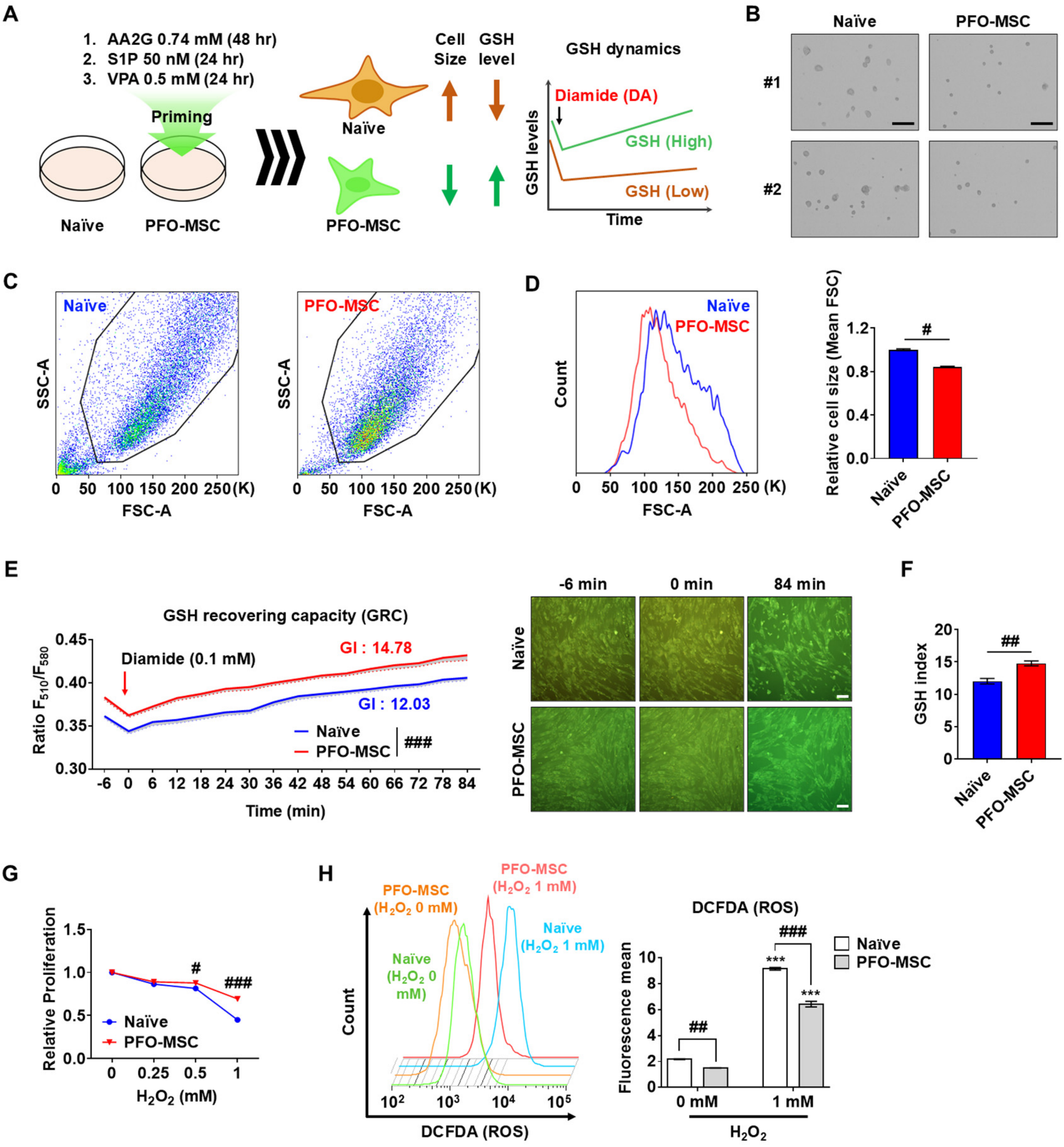

Figure S8. Enhanced core functions of PFO-MSCs

(A) Overview of the PFO procedure and GSH recovering capacity (GRC) assay. Primitive adult tissue-derived MSCs, which are characterized by their small size and high GSH dynamics, can be enriched by propagation with ascorbic acid-2 glucoside (AA2G) and further stimulation with a low concentration of sphingosine-1 phosphate (S1P) and valproic acid (VPA) by the PFO procedure, as previously reported[1,2]. **(B–D)** Microscopic **(B)** and flow cytometric **(C and D)** analyses measuring the size of normal (naïve) hUC-MSCs and PFO-MSCs (magnification, 200×; scale bar, 100 μm). **(E)** Real-time monitoring of GRC and basal GSH levels in naïve MSCs and PFO-MSCs upon exposure to 100 μM diamide (arrow). **(F)** The GSH dynamics of each sample were quantified based on the initial F_{510}/F_{580} fluorescence ratio (for baseline total GSH) and slope after diamide treatment (for GRC), as previously described[1,3]. **(G and H)** Cell viability **(G)** and reactive oxygen species (ROS) levels **(H)** in naïve hUC-MSCs and PFO-MSCs after exposure to the indicated concentration of H_2O_2 for 2 h. **(H)** Flow cytometry analysis by staining 2',7'-dichlorodihydrofluorescein diacetate (DCFDA) was used to detect the intracellular ROS production in the indicated cells. All quantitative data are presented as the mean ± SEM (*n* = 5). Data were analyzed by the non-parametric Mann-Whitney test **(D and F)** or two-way **(E, G, and H)** ANOVA with the Bonferroni post-hoc comparison (#p<0.05, ##p<0.01, ###p<0.001). The exact p-values and number of replicates are specified in **Data S1**.

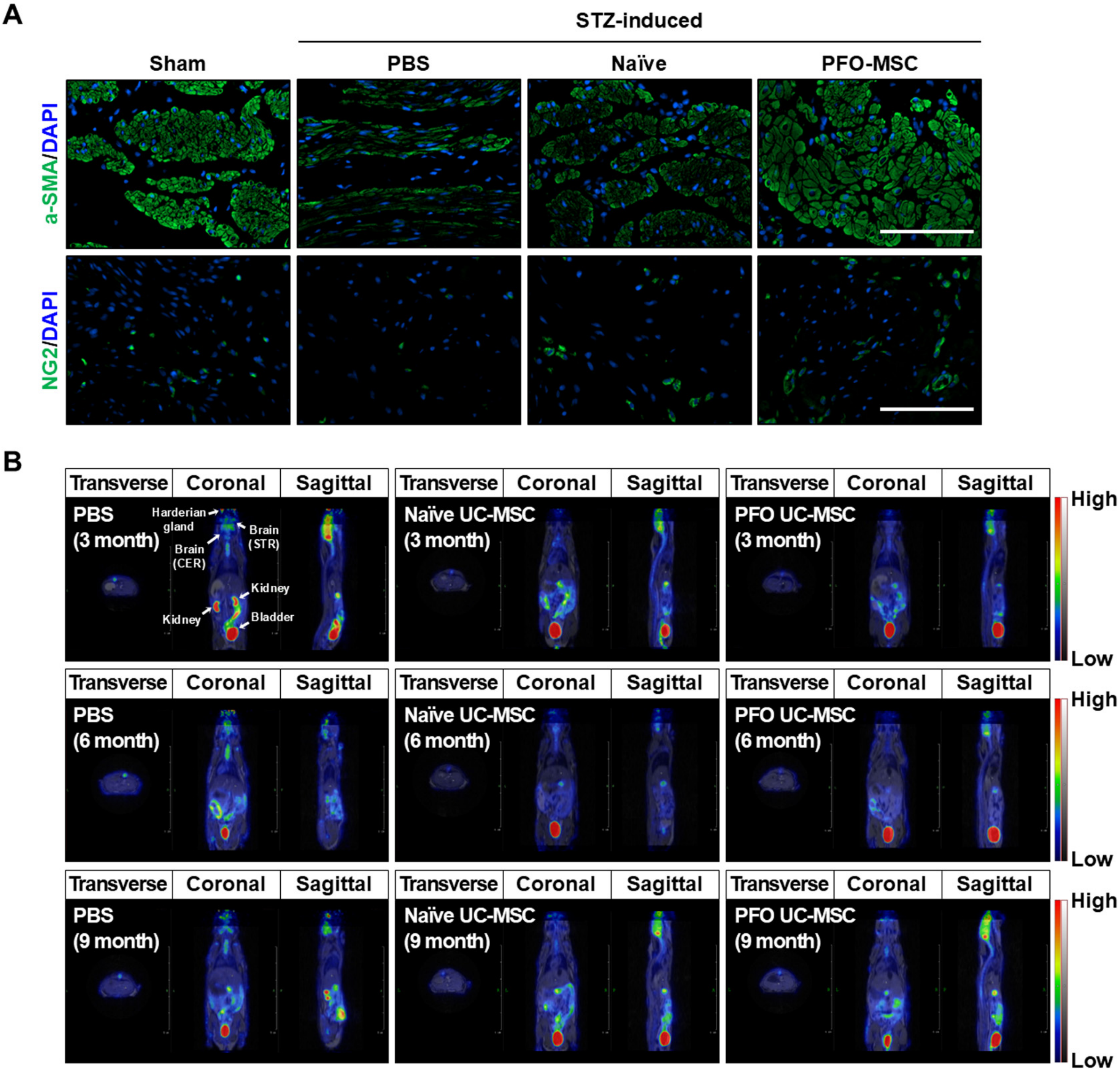

Figure S9. Therapeutic and long-term saftey outcomes of transplanted PFO-MSCs.

(A) Representative images (magnification, 400×; scale bar, 200 μm) of immunofluorescence staining of muscle (α-SMA, upper panel) and pericyte (NG2, lower panel) markers in bladder

sections of the indicated groups. Nuclei were counterstained with DAPI (blue). **(B)** Longitudinal micro-positron emission tomography/magnetic resonance imaging (μ-PET/MRI) imaging for 9-months after injection. The transverse, coronal, and satittal views of fused MRI (T1 GRE EX) and PET images (15 min scan after 2-[^{18}F]-fluoro-2-deoxyglucose (FDG) injection) of NOD/ShiLtJ-*Prkdc*^{em1AMC}*Il2rg*^{em1AMC} (NSGA) mice at 3, 6, and 9 months after injection of phosphate-buffer saline (PBS) vehicle (left panel) or transplantation of 1×10^5 naïve (middel panel) or PFO procedured (right panel) hUC-MSCs (*n* = 5). Notably, only background signal for uptake of [^{18}F]-FDG was detected, and no other [^{18}F]-FDG uptake characteristics of tumors were observed in any NSG animal injected with hUC-MSCs or PBS vehicle.

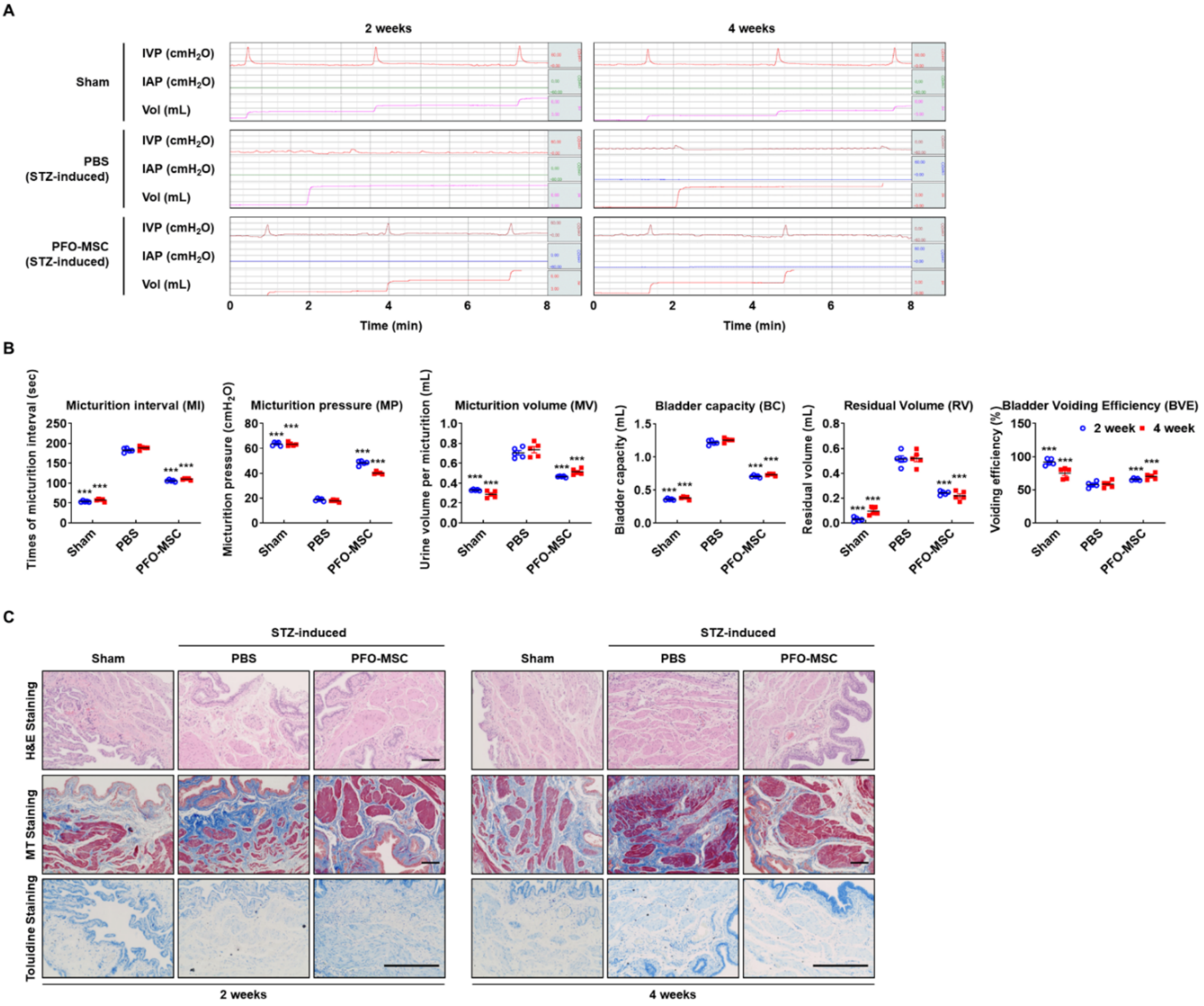

Figure S10. Long-term therapeutic effects of PFO-MSCs in diabetic DUA

(A and B) Representative results of awake cystometry **(A)** and quantitative bladder voiding data at 2 or 4 weeks after injection of 5.0×10^5 naïve hUC-MSCs or PFO-MSCs into STZ-DUA rats. The quantitative results are shown as a dot plot of mean ± SEM (*n* = 5). Data were analyzed by a two-way ANOVA with the Bonferroni post-hoc comparison (***p<0.001 relative to the PBS group). **(C)** Histological analysis including i) H&E staining (magnification, 100×; scale

bar, 100 μm), ii) Masson's trichrome staining (magnification, 100×; scale bar, 100 μm), and iii) Toluidine blue staining (magnification, 400×; scale bar, 200 μm). Nuclei were stained with Mayer's hematoxylin.

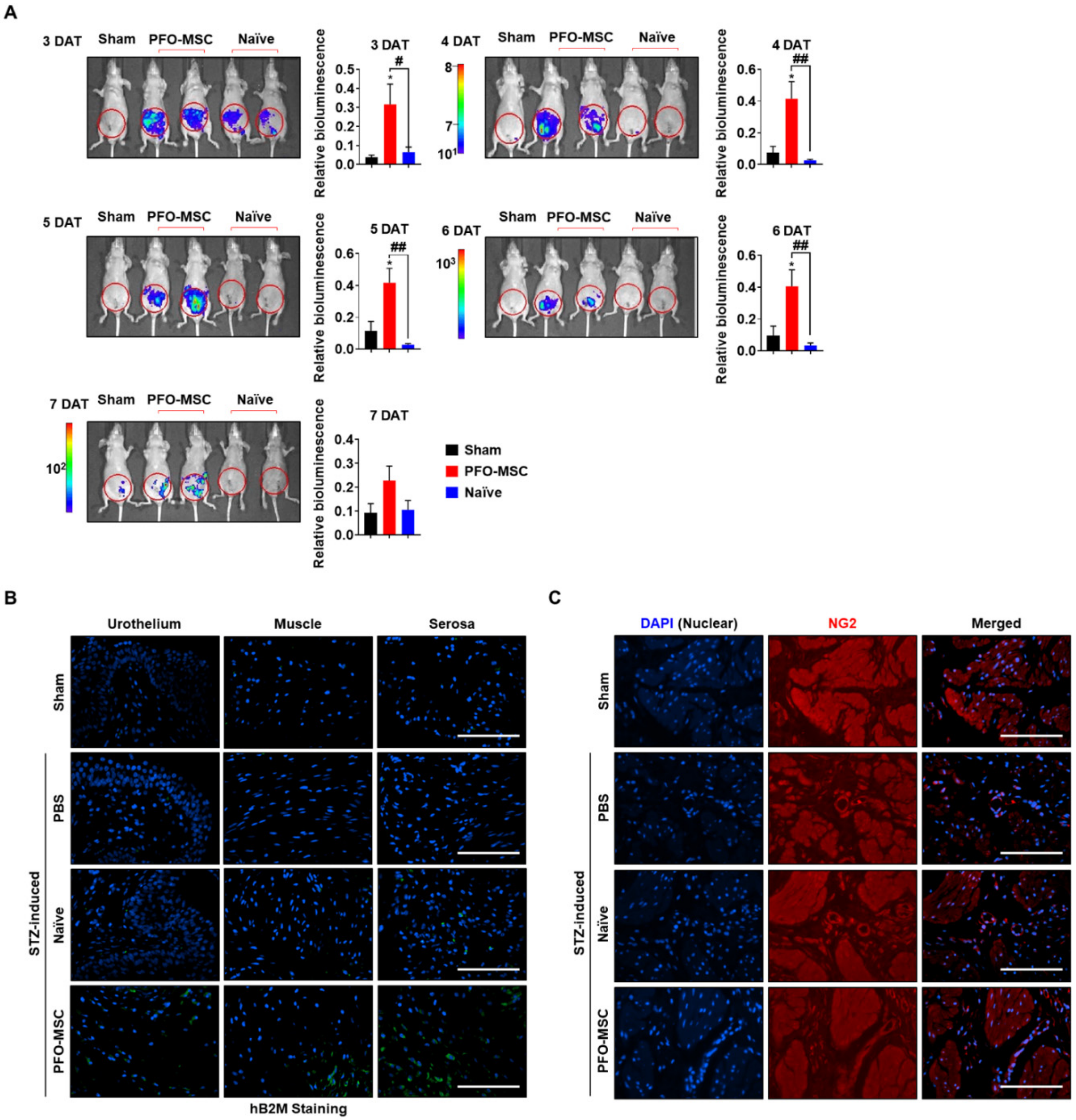

Figure S11. Engraftment analysis of transplanted PFO-MSCs

(A) IVIS optical imaging analysis of bioluminescence activity of Nano-lantern^{+} naïve hUC-

MSCs or PFO-MSCs (1×10^5) in a live BALB/c-nu immunocompromised mouse during 7 DAT. Before administration of the indicated Nano-lantern$^+$ MSCs, diabetic DUA was induced by STZ injection. Relative bioluminescence signals at the indicated DAT are presented as the relative values to 1 DAT value at the PFO-MSC group. Quantitative results are presented as the mean ± SEM, are shown to the right of the representative IVIS images for each DAT (*n* = 5). *p<0.001, ***p<0.001 compared with the sham group; #p<0.05 and ##p<0.01, one-way ANOVA with the Bonferroni post-hoc test. Representative images of bioluminescence resonance energy transfer activities were obtained 15 minutes after intraperitoneal injection of 150 μg/mL coelenterazine (200 μL), a substrate of Renilla luciferase. Nano-lantern: a chimera of enhanced Renilla luciferase and Venus fluorescent protein. **(B)** Representative images of hB2M in the indicated bladders (magnification, 400×; scale bar, 200 μm). The engrafted hB2M$^+$ PFO-MSCs were primarily located at the injection site between the muscle and serosal layers of the bladder, with a few observed in the lamina propria, and were rarely detected in the urothelial layer. The hB2M$^+$ cells were hardly detected in animals without transplantation of MSCs. **(C)** Representative fluorescence micrographs of NG2 (red), a pericyte marker, in bladder tissues of STZ-DUA rats at 1 week after injection of PBS (vehicle) or the indicated MSCs (magnification, 400×; scale bar, 200 μm). Nuclei were stained with DAPI (blue). In our previous report for a preclinical study of interstitial cystitis/bladder pain syndrome (IC/BPS), an intractable bladder voiding dysfunction characterized by loss of urothelium integrity and a chronic inflammatory response in the bladder[4], the engrafted MSCs gradually integrate as perivascular cells through interactions with the vasculature of the bladder. In present diabetic DUA model, compared with naïve hUC-MSCs, PFO-MSC-injected animals displayed more cells expressing NG2 proteoglycan, a marker of pericytes, and NG2$^+$ cells were predominantly observed around muscle fibers.

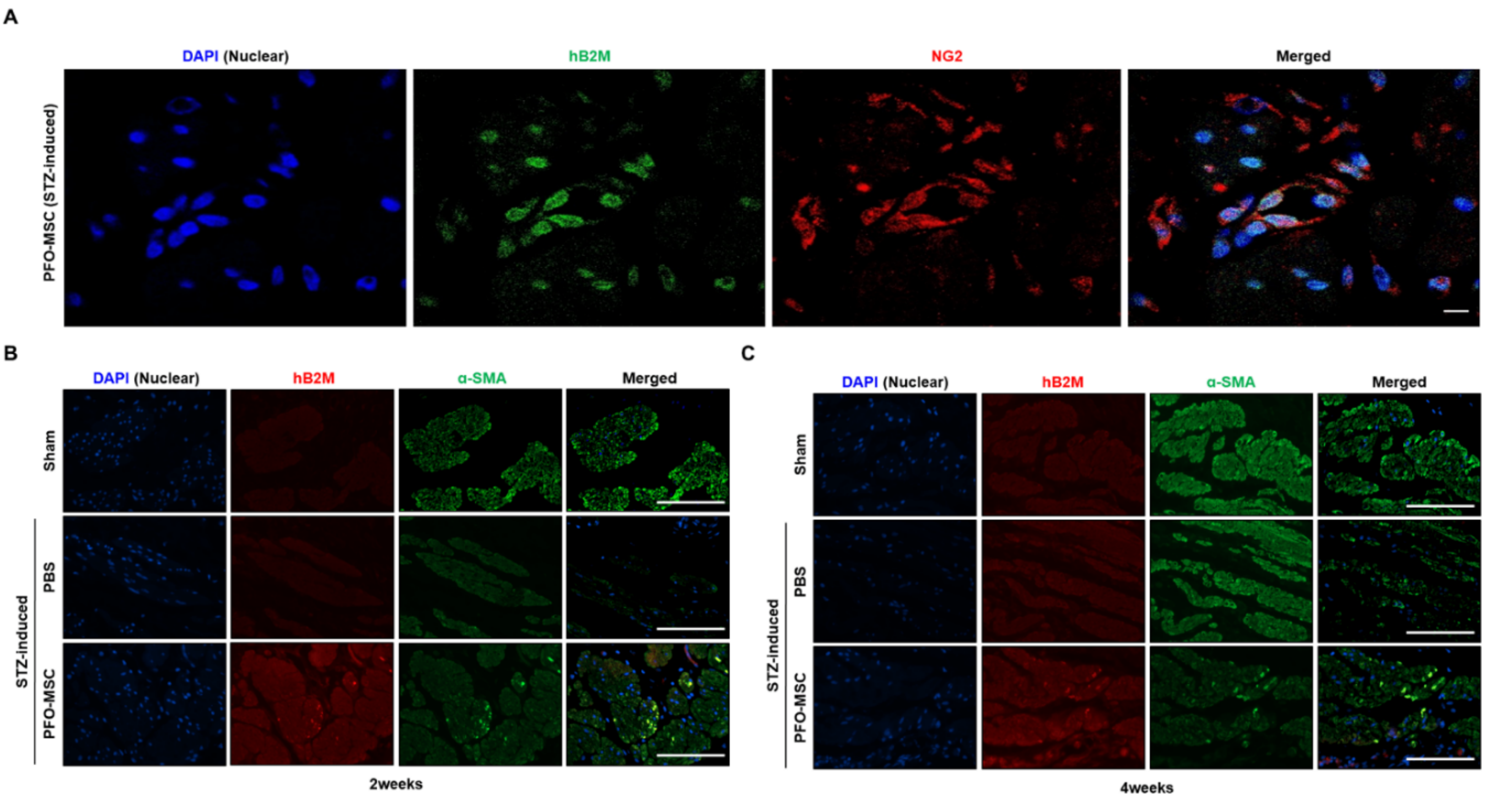

Figure S12. Integration of engrafted PFO-MSCs as myofiber cells

(A) Representative confocal micrographs of co-staining of hB2M (red) and NG2 (green), a pericyte marker protein in bladder sections of STZ-DUA rats at 1 weeks after transplantation of PFO-MSCs (magnification, 1000×; scale bar, 10 μm). Co-staining of hB2M and NG2 indicated that a notable portion of $NG2^{+}$ cells around muscle fibers also expressed hB2M. **(B and C)** Representative immunofluorescence micrographs of co-staining of hB2M (red) and α-SMA, a muscle marker protein in the indicated bladder sections of STZ-DUA rats at 2 **(B)** and 4 **(C)** weeks after transplantation (magnification, 400×; scale bar, 200 μm).

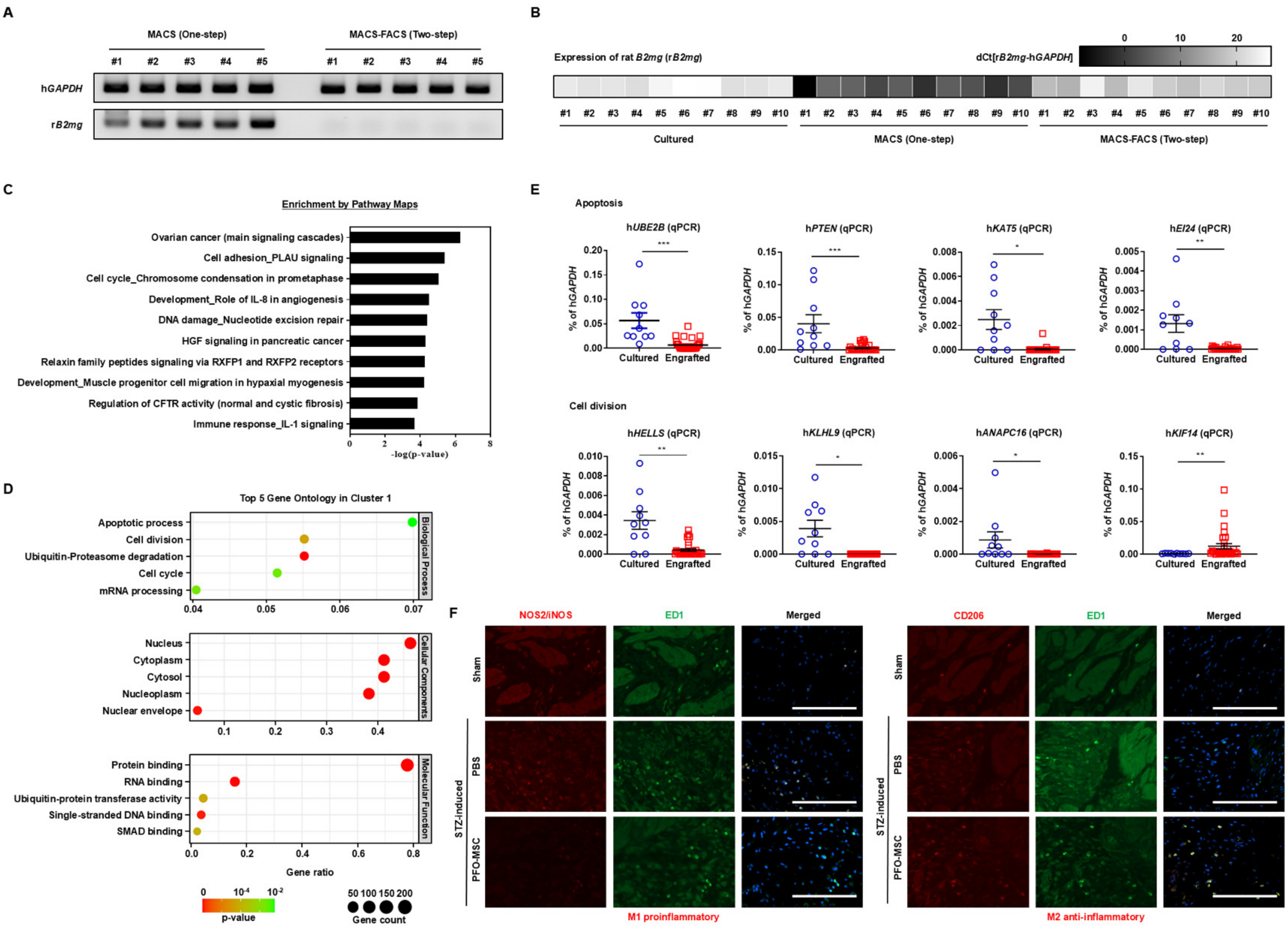

Figure S13. Isolation and single-cell transcriptome analysis of engrafted PFO-MSCs

Single-cell transcriptome profiling to reveal the molecular characteristics of engrafted PFO-MSCs within a pathological microenvironment. The rare cells that engrafted in diabetic DUA were purified via magnetic-activated cell sorting (MACS) followed by fluorescence-activated cell sorting (FACS) after transplantation of GFP^{+} PFO-MSCs labeled with Resovist, a clinically approved superparamagnetic iron oxide agent. This method not only enhances the purity of isolated engrafted cells with little contamination from host cells, but also circumvents the experimental challenges associated with extended assay times and reductions in yield and

purity when directly employing FACS to sort cells with low frequencies[4]. Cells purified through the two-stage MACS-FACS procedure were individually transferred to a single well of a 96-well plate, and single-cell transcriptome libraries were constructed following the T7-primed amplification protocol[5]. The single-cell libraries were prepared from cultured PFO-MSCs (Cultured_#1–5) or Resovist^{+}/GFP^{+} engrafted cells (Eng_#1–10) isolated from animals with diabetic DUA at 7 DAT.

(A and B) Initial screening of these libraries was conducted to identify any host cell contamination, which detected little expression of rat-specific β2 microglobin (r*B2mg*) mRNA. **(A)** Representative results of gel electrophoresis showing expression of human *GAPDH* (h*GAPDH*) and r*B2mg* housekeeping genes following one-step MACS or two-step MACS-FACS procedures. **(B)** Heatmap analysis of Ct values from RQ-PCR analysis of h*GAPDH* and r*B2mg* expression using the indicated single-cell cDNA libraries. Expression levels are shown as ΔCt values relative to h*GAPDH* expression. **(C)** The ten most highly enriched pathway maps identified by MetaCore analysis of engrafted cells. Notably, these differences were characterized by alterations in expression of genes related to muscle progenitor cells, hepatocyte growth factor (HGF) signaling, cell adhesion, and immune responses. **(D)** The five most highly enriched GO terms representing cluster 1 genes, which were consistently downregulated in engrafted cells. **(E)** qPCR analysis of a subset of cluster 1 genes related to apoptosis and cell division in engrafted (Engrafted, n = 30) and cultured (Cultured, n = 10) single-cell cDNA libraries. Gene expression is presented as the percentage relative to h*GAPDH* expression and shown as a dot plot of mean ± SEM (non-parametric Mann-Whitney test, *p<0.05, **p<0.01, ***p<0.001). **(F)** Immunostaining of NOS2/iNOS (red)-positive pro-inflammatory macrophages (left panel) and CD206 (red)-positive anti-inflammatory macrophages (right panel) together with ED1 (green), a pan-specific macrophage marker

protein (magnification, 400×; scale bar, 200 μm). Notably, injection of PFO-MSCs induced infiltration of M2 macrophages, which mediate an anti-inflammatory response, based on the findings that ED1-expressing macrophages in rats with diabetic DUA transplanted with PFO-MSCs primarily expressed CD206, an M2 subtype marker, while NOS2/iNOS, an M1 subtype marker, was prevalent in bladder tissue in the STZ-DUA group

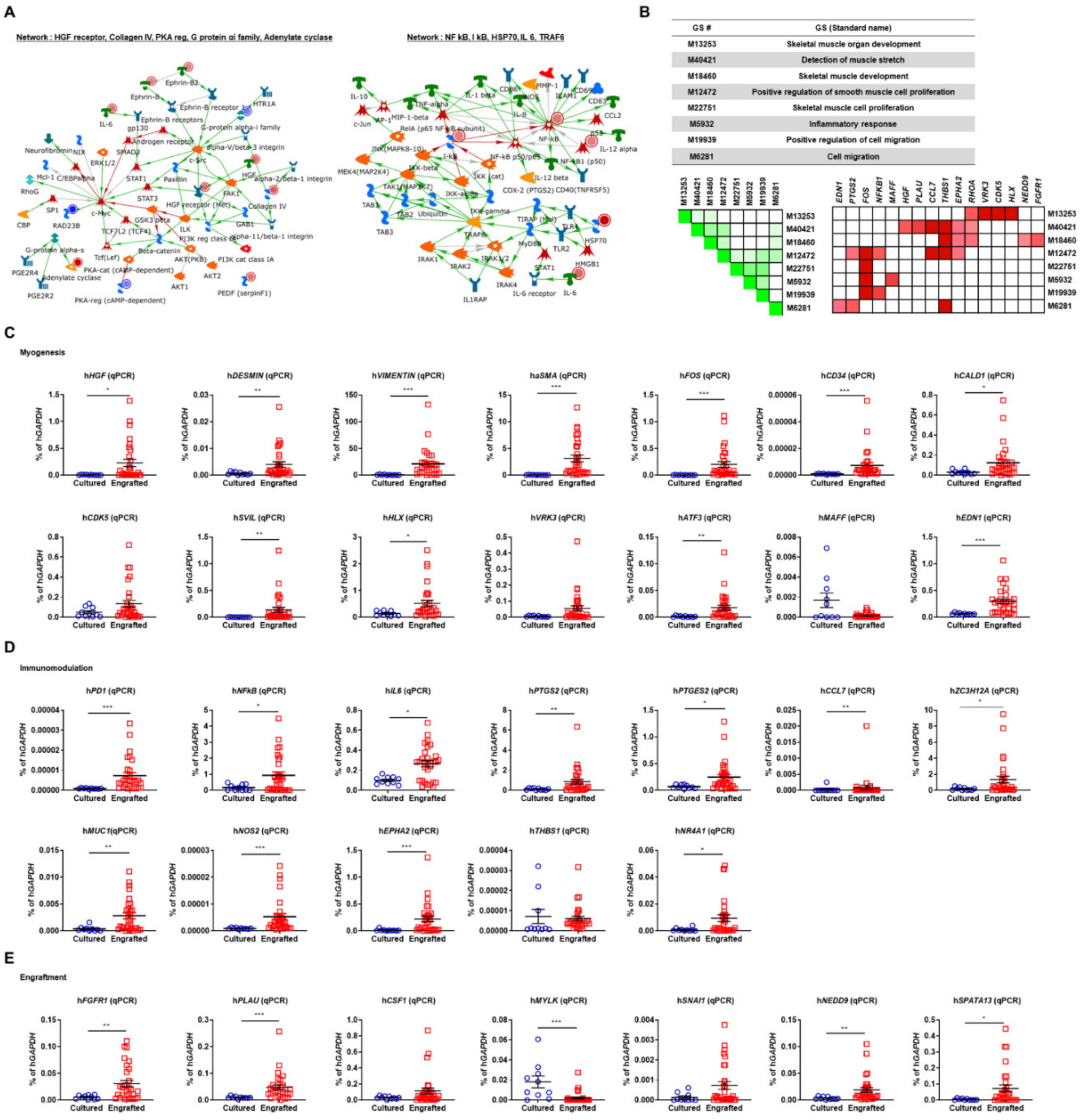

Figure S14. Gene expression analysis of engrafted cells in diabetic bladders

(A) Representative enriched gene networks related to skeletal muscle tissue development (left panel) and immune or inflammatory responses (right panel) of engrafted cells determined by MetaCore analysis. The networks are illustrated by overlaying the experimental values as fold changes in engrafted versus cultured cells. Upregulated and downregulated genes are indicated in red and blue, respectively. **(B)** GSEA leading-edge analysis using the gene sets enriched in engrafted cells. Compared with libraries of cultured cells, those of engrafted cells showed distinct expression of genes related to skeletal muscle tissue development (e.g., *HGF*, *RHOA*, *FOS*, and *MYLK*) and immune or inflammatory responses (e.g., *CD274*/*PD-L1*, *IL6*, *TRAF6*, and *NFkB*). Among them, HGF and PD-L1, representing muscle regeneration and immunomodulatory processes, respectively, were identified in GSEA and MetaCore analyses. **(C–E)** qPCR analysis validating the engrafted (Engrafted, n = 30) and cultured (Cultured, n = 10) single-cell cDNA libraries. Expression of genes related to myogenesis **(C)**, immunomodulation **(D)**, and engraftment **(E)** processes is presented as percentages relative to human *GAPDH* (h*GAPDH*) expression and shown as a dot plot of mean ± SEM. Statistical significance was examined by the non-parametric Mann-Whitney test (*p<0.05, **p<0.01, ***p<0.001).

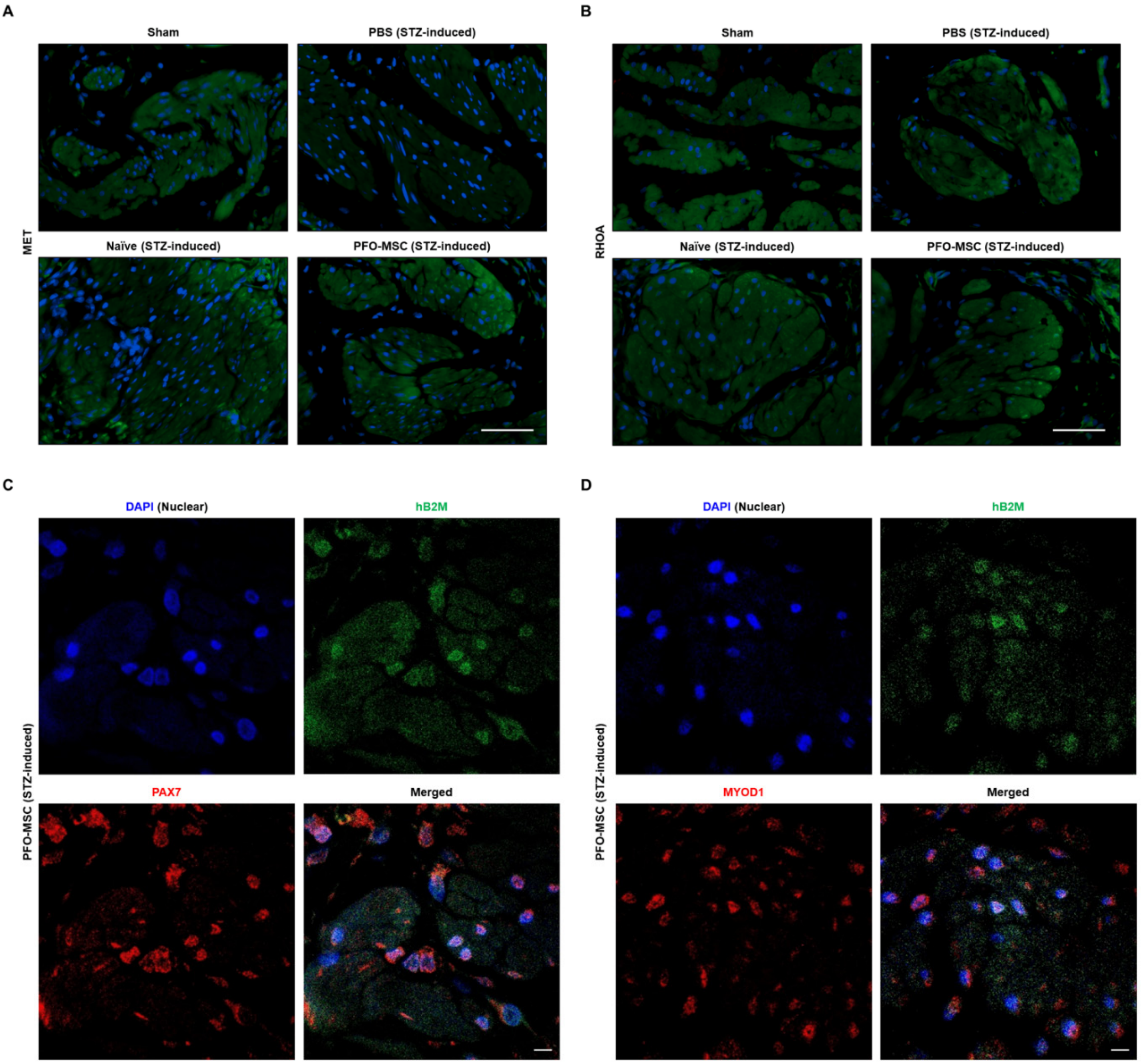

Figure S15. Immunofluorescence analysis of engraftment of PFO-MSCs

(A and B) Representative fluorescence micrographs (magnification, 400×; scale bar, 200 μm) of MET **(A)** and RHOA **(B)** in rat bladder sections from the indicated groups. In particular, the hB2M$^+$ cells, situated within or in close proximity to muscle bundles, robustly expressed MET

and RHOA proteins. **(C and D)** Representative confocal fluorescence micrographs (magnification, 1000×; scale bar, 10 μm) of co-staining of myogenic markers (red), including PAX7 **(C)** and MYOD **(D),** and hB2M (green) in bladder tissues from the indicated groups. Nuclei were stained with DAPI (blue). The hB2M^{+} cells within muscle bundles expressed PAX7 and MYOD1muscle marker proteins. Collectively, these results indicate that PFO-MSCs directly contribute to regeneration of smooth muscle cells in diabetic DUA.

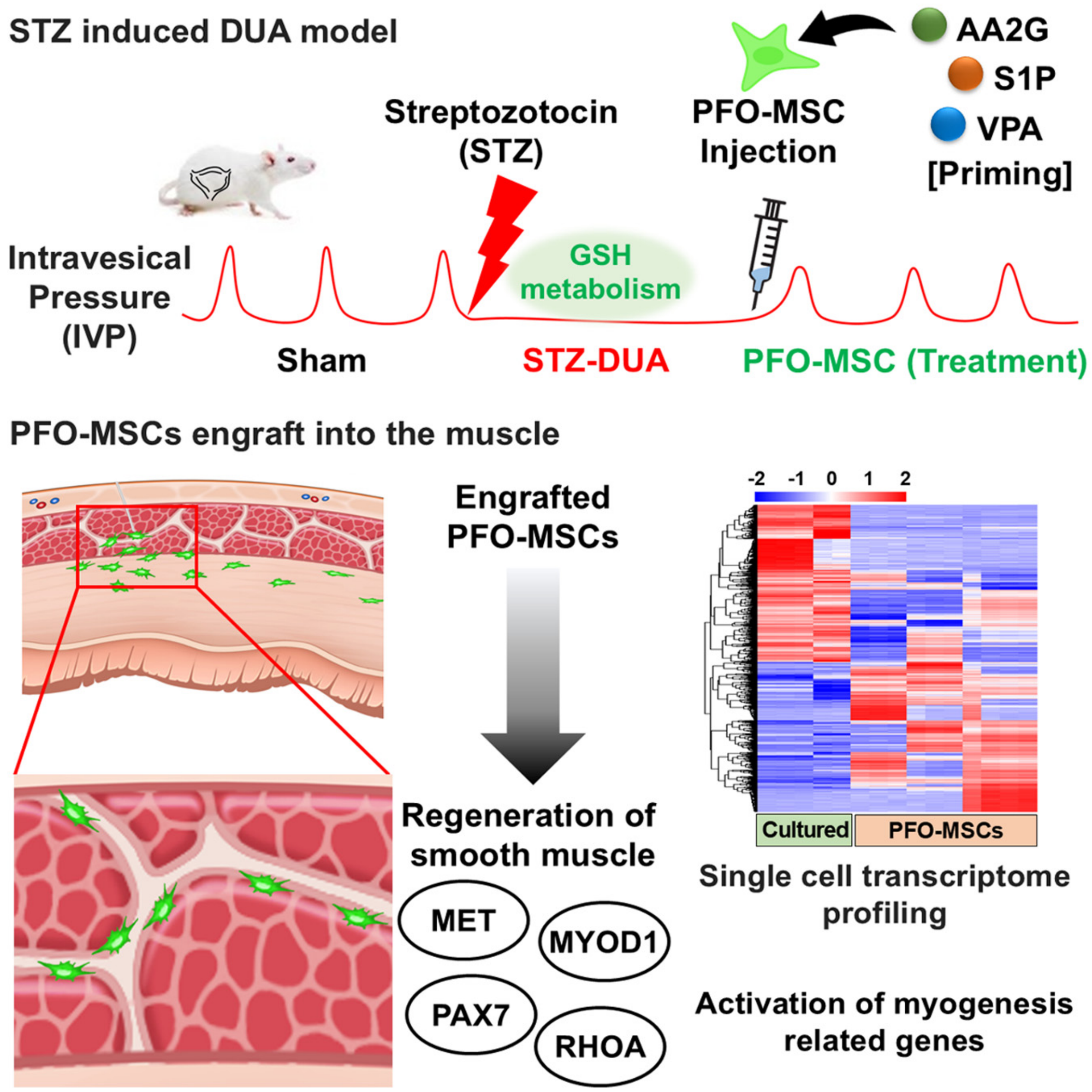

Figure S16. Graphic abstract

This study utilizes transcriptomic analysis of bladder tissue from a preclinical stem cell therapy investigation, revealing the tissue injury related to glutathione dynamics as a pivotal factor in diabetes-induced DUA. By presenting experimental evidence, it advocates for the use of safe umbilical cord-derived stem cells (PFO-MSC) with heightened glutathione dynamics for DUA treatment. Single-cell transcriptome analysis highlights the enhanced *in vivo* engraftment and

sustained viability of PFO-MSCs, showcasing their direct differentiation into muscle progenitors. Furthermore, the study underscores the significance of the HGF–MET cascade in this therapeutic process.

SUPPLEMENTARY TABLE LEGENDS; Provided by a separate Excel file.

Table S1. GSEA of transcriptomes between STZ-DUA and sham groups, Related to Figure 1

(A) Genesets defining in comparison of transcriptomes between STZ-DUA and sham groups. The 10 top-scoring genesets enriched in the STZ-DUA group and the 10 top-scoring genesets enriched in the sham group are listed in upper and lower tables, respectively, based on GSEA of transcriptome. The genesets are listed according to normalized enrichment score (NES). GSEA, geneset enrichment analysis; ES, enrichment score; NOM p-val, nominal p-value; FDR, false discovery rate. **(B)** Genesets defining in comparison of transcriptomes between STZ-DUA and sham groups. The 10 top-scoring gene ratio in the cluster-3 genes, respectively, based on KEGG pathway analysis of transcriptome. The genesets are listed according to gene ratio. NOM p-val, nominal p-value; FDR, false discovery rate.

Table S2. Identification of features for the STZ-DUA, Related to Figure S2

Leading-edge genes characterizing the STZ-DUA group. List of leading-edge genes enriched in comparison of transcriptomes between STZ-DUA and M-MSC groups in GSEA using a different source of geneset libraries.

Table S3. Identification of features for the engrafted PFO-MSCs, Related to Figure S14

Leading-edge genes characterizing the engrafted PFO-MSCs group. List of leading-edge genes enriched in the engrafted PFO-MSCs group in GSEA using a different source of geneset libraries.

Table S4. The putative biomarkers characterizing the engrafted PFO-MSCs in the diabetic DUA, Related to Figure 4

Quantitative PCR analysis of biomarkers for the engrafted PFO-MSCs. Fold changes and p-values of quantitative PCR (qPCR) analyses are presented based on the expression of each gene presented as delta Ct (dCt) value or percentage of GAPDH. The bubble pot results are shown in **Figure 4A**.

Table S5. Oligonucleotides, Related to STAR Methods

Primer sequences for qPCR assay

KEY RESOURCES TABLE

REAGENT or RESOURCE	SOURCE	IDENTIFIER
Antibodies		
aSMA	Abcam	Cat# ab7817, RRID:AB_262054
CD206	Abcam	Cat# ab64693, RRID: AB_1523910
ED1	Santa cruz	Cat# sc-20060, RRID: AB_627158
GCLC	Abcam	Cat# ab190685, RRID:AB_2889925
GCLM	Abcam	Cat# ab81445, RRID:AB_1860504
GPX2	Abcam	Cat# ab137431, RRID:NA
GSR	Abcam	Cat# ab128933, RRID:AB_11142591
NOS2/iNOS	Abcam	Cat# ab3523, RRID: AB_303872
MET	Santa cruz	Cat# sc-514148, RRID:NA
MYOD	Cell Signaling Technology	Cat# 13812, RRID:AB_2798320
NG2	Santa cruz	Cat# sc-53389, RRID:AB_784821
PAX7	Invitrogen	Cat# PA1-117, RRID:AB_2539886
PD-L1	Abcam	Cat# ab205921, RRID:AB_2687878
RHOA	Santa cruz	Cat# sc-101058, RRID:AB_628218
NOXA1	Santa cruz	Cat# sc-398873, RRID:NA
NOXO1	Santa cruz	Cat# sc-390927, RRID:NA
hB2M	Santa cruz	Cat# sc-80668, RRID:AB_1119512
Alexa488 anti-rabbit	Thermo Fisher Scientific	Cat# A-11008, RRID:AB_143165
Alexa488 anti-mouse	Thermo Fisher Scientific	Cat# A-11001, RRID:AB_2534069
Alexa546 anti-mouse	Thermo Fisher Scientific	Cat# A-11060, RRID:AB_2534107
DAPI	Sigma-Aldrich	Cat# D9542, RRID:NA
Bacterial and Virus Strains		
pCDH-CMV lentiviral vector	Gifted from Kazuhiro Oka (Addgene)	Cat# 72265, RRID:Addgene_72265
Chemicals, Peptides, and Recombinant Proteins		
DMEM–low-glucose medium	HyClone	Cat# SH30243.01

FBS	Hyclone	Cat# SH30084.03
L-Glutamine	Corning	Cat# 26-005-CI
HEPES	Corning	Cat# 25-060-CI
MEM nonessential amino acid solution	Corning	Cat# 25-025-CIR
Penicillin/streptomycin solution	Corning	Cat# 30-002-CI
Human epidermal growth factor (EGF)	Sigma-Aldrich	Cat# E9644
Basic fibroblast growth factor (bFGF)	ProSpec	Cat# CYT-218
Long-R3 insulin-like growth factor-1 (IGF-1)	ProSpec	Cat# CTY-022
Ascorbic acid	Sigma-Aldrich	Cat# A4544
AA2G	Sigma-Aldrich	Cat# SMB00390
Sphingosine-1-phosphate (S1P)	Cayman	Cat# 62570
Valproic Acid (VPA)	Sigma-Aldrich	Cat# P4543
EGM2-MV	Lonza	Cat# CC-3156
Collagen type I	Sigma-Aldrich	Cat# C7661
NAC	Sigma-Aldrich	Cat# A9165
Accutase	Gibco	Cat# A1110501
RBC Lysis Solution	QIAGEN	Cat# 158902
4% Paraformaldehyde	Sigma-Aldrich	Cat# P6148
Diamide	Sigma-Aldrich	Cat# D3648
RNeasy purification kit	QIAGEN	Cat# 74104
Streptozotocin	Sigma-Aldrich	Cat# S0130
Lipofectamine 2000	Invitrogen	Cat# 11668500
Critical Commercial Assays		
Taqman Reverse Transcription Reagents	Applied Biosystems	Cat# N8080234
Block-iT Lentivrial RNAi Expression System	Invitrogen	Cat# K494400
Lenti-X Concentrator kit	Clontech	Cat# 631232
Cell Viability Assay Kit EZ-CYTOX	Daeil Lab	Cat# EZ-3000
QuantSeq 3’ mRNA-Seq Library Prep Kit	Lexogen	N/A
OxyBlot Protein Oxidation Detection Kit	Millipore	Cat# S7450
DCFDA / H2DCFDA - Cellular ROS assay kit	Abcam	Cat# ab113851
RNeasy Mini Kit	QIAGEN	Cat# 74106
Deposited Data		
Transcriptome datasets for STZ-DUA	This paper	GSE263322
Transcriptome datasets for PFO-MSCs	This paper	GSE263323
Experimental Models: Cell Lines		
hES-MSCs	Hong et al., 2014	N/A
Human UC-MSCs	Lim et al., 2017	N/A
293FT	Thermo Fisher Scientific	Cat# R70007
Experimental Models: Organisms/Strains		
BALB/c-nu (CAnN.Cg-Foxn1nu/CrlCrlj) Immunodeficient Mice	ORIENT BIO	03 CRJ (Charles River Japan)
Sprague-Dawley rats	ORIENT BIO	N/A
NOD/ShiLtJ-*Prkdc*^{em1AMC}*Il2rg*^{em1AMC} (NSGA)	JA BIO	Cat# NSG_A
Oligonucleotides		

Primers for qPCR, see **Table S5**	This study	N/A
Software and Algorithms		
GraphPad Prism 7.6.5	GraphPad Software	N/A
MetaCore	Clarivate Analytics	N/A
GSEA	Broad Institute	N/A
ExDEGA	Ebiogen Inc.	N/A
AcqKnowledge 3.8.1 software	Biopac Systems	N/A
Living Imaging 4.4 software	PerkinElmer	N/A
Other		
MP150 data acquisition system	Biopac Systems	N/A
BD FACSCanto II flow cytometer	BD Biosciences	N/A
ARIAIII Flow Cytometer System	BD Biosciences	N/A
EVOS FL Color Imaging System	Life Technologies	Cat# AMF4300
ZEISS LSM710 confocal microscope system	Carl Zeiss	N/A
IVIS Spectrum Preclinical In vivo Imaging Syste m	PerkinElmer	N/A

SUPPLEMENTARY METHODS

Culture and in vitro characterization of MSCs

hUC-MSCs were cultured in low-glucose Dulbecco's Modified Eagle's Medium (DMEM) containing 10% heat-inactivated fetal bovine serum (FBS), 5 ng/mL human EGF (Sigma-Aldrich, St. Louis, MO, USA), 10 ng/mL basic fibroblast growth factor, and 50 ng/mL long-R3 insulin-like growth factor-1 (ProSpec, Rehovot, Israel), following established protocols[1,2]. To ensure functionality, all MSCs utilized in this study underwent fewer than seven passages and were maintained at 37°C in a humidified atmosphere with 5% CO_2.

For the PFO procedure, hUC-MSCs were seeded at a density of 7×10^4 cells/mL and cultured in medium supplemented with 0.74 mM AA2G (Sigma-Aldrich) for the specified duration. One day before functional assessment, 50 nM S1P (Sigma-Aldrich) and 0.5 mM VPA (Sigma-Aldrich) were added to the culture medium with 0.74 mM AA2G. *In vitro* evaluations of PFO-MSCs, encompassing cell size, GRC, levels of reactive oxygen species (ROS), and resistance to oxidative stress, were conducted, as previously described[6,7]. Flow cytometric data were analyzed using FlowJo software 7.6.5 (FlowJo, LLC, Ashland, OR, USA).

M-MSCs differentiated from H9 hESCs were sustained in EGM2-MV medium (Lonza, San Diego, CA, USA) on plates coated with rat tail collagen type I (Sigma-Aldrich) in a humidified atmosphere with 5% CO_2 at 37°C, following previously described procedures[8,9].

Diabetic DUA animal model and treatment with MSCs, NAC, or their combination

Female Sprague-Dawley rats aged 8 weeks were employed. Type I diabetes was induced using STZ (Sigma Chemical Company, St. Louis, MO, USA). Following an overnight fast, rats were intraperitoneally injected with STZ (50 mg/kg) dissolved in a 0.1 M citrate acid buffer solution

(pH 4.5). The non-diabetic control group was injected with an equivalent volume of citrate buffer solution. Blood glucose levels were assessed 72 hours later via tail prick samples, and rats with levels exceeding 200 mg/dL (16.7 mmol/L) were identified as diabetic and included in subsequent studies. Three weeks post-diabetes induction, diabetic rats were anesthetized with 0.2 mL tiletamine (Zoletil1; Virbac Laboratories, Carros, France) to evaluate the therapeutic efficacy of MSCs or NAC alone as well as their combination. Human MSCs resuspended in 200 µL of phosphate-buffered saline (PBS) were directly injected into the outer layer (serosa) of the anterior bladder wall, as previously described[4,10]. For the non-diabetic and untreated diabetic groups, PBS was injected instead of stem cells. For NAC monotherapy, 100 or 200 mg/kg NAC (Sigma-Aldrich) was administered daily via intraperitoneal injection for 5 days, followed by a 2-day rest period. For combination therapy, following M-MSC administration, 500 µL of PBS with or without 200 mg/kg NAC was intraperitoneally administered for 5 days, followed by a 2-day rest period. The therapeutic outcomes were assessed via awake cystometry and histological analysis at 1 week (short-term) or 2 and 4 weeks (long term) after administration of MSCs or NAC alone and their combination.

Evaluation of bladder function and tissue preparation

Cystometric assessment was conducted in conscious, unrestrained animals placed in metabolic cages. Three days before cystometry, intravesical pressure (IVP) and intra-abdominal pressure (IAP) were measured. The urethra was accessed using a PE-50 catheter (Clay Adams, Parsippany, NJ, USA) attached to a pressure transducer (Research Grade Blood Pressure Transducer; Harvard Apparatus, Holliston, MA, USA) and a microinjection pump (PHD22/2000 pump, Harvard Apparatus). Voiding volumes were monitored through a fluid collector connected to a force displacement transducer (Research Grade Isometric Transducer,

Harvard Apparatus) as normal saline was infused into the bladder at a rate of 0.4 mL/min. IVP, IAP, and voiding volume were continuously recorded using Acq Knowledge 3.8.1 software and an MP150 data acquisition system (Biopac Systems, Goleta, CA, USA) at a sampling rate of 50 Hz. The analysis utilized mean values from three reproducible voiding cycles of each animal. Non-voiding contractions were identified when IVP increments exceeded 15 cm H_2O from baseline without expelled urine. Bladder pressure, MP, MV, and RV were determined. The MI was determined as the duration between micturition contraction cycles. BC equaled MV + RV. BVE was calculated as 100 × MV/BC. Mean values from three reproducible micturition cycles were assessed in five animals per group. Following voiding function evaluation, rat bladders were collected. Half of each bladder was cryopreserved in liquid nitrogen for RNA isolation, while the remaining half was fixed in 4% buffered formalin and embedded in paraffin for histological examination or immunohistochemical staining.

Histological analysis and immunofluorescence staining

Following fixation in 4% paraformaldehyde for 24 hours, bladders were immersed in paraffin, sectioned into 3 μm slices using a microtome, affixed onto slides, and subjected to hematoxylin and eosin (H&E) staining. Mast cell infiltration and fibrosis were assessed by Toluidine blue staining (Toluidine blue-O; Daejung Chemicals & Metals, Seoul, Korea) and Masson's trichrome staining (Junsei Chemical, Tokyo, Japan), respectively. Carbonyl groups introduced into proteins by the oxidative reaction were detected using an OxyBlot Protein Oxidation Detection Kit (Millipore, Billerica, MA, USA), according to the manufacturer's instruction.

Expression of the indicated proteins in bladders was investigated by immunofluorescence staining using antibodies specific to GPX2 (ab137431; Abcam, Cambridge, UK), GSR (ab128933; Abcam), GCLC (ab190685; Abcam), α-SMA (ab7817;

Abcam), NG2 (sc-53389; Santa Cruz Biotechnology, Santa Cruz, CA), NOS2/iNOS (ab3523; Abcam), ED1 (sc-20060; Santa Cruz Biotechnology), MET (sc-514148; Santa Cruz Biotechnology), PAX7 (PA1-117; Invitrogen, Waltham, MA, USA), MYOD (#13812; Cell Signaling Technology, Danvers, MA, USA), RHOA (sc-418; Santa Cruz Biotechnology), PD-L1 (ab205921; Abcam), NOXA1 (sc-398873; Santa Cruz Biotechnology) and NOXO1 (sc-390927; Santa Cruz Biotechnology). The engraftment and cellular fate of MSCs transplanted into bladders were evaluated by co-staining of bladders with antibodies against hB2M (sc-80668; Santa Cruz Biotechnology) and the indicated cell fate marker proteins. Alexa Fluor 488-conjugated (A11001) and Alexa Fluor 564-conjugated (A11010) anti-mouse and anti-rabbit secondary antibodies (Molecular Probes, Grand Island, NY, USA) were used. Nuclei were counterstained with 4',6-diamino-2-phenylindole (DAPI; D9542, Sigma-Aldrich). Images were acquired using an inverted fluorescence microscope (EVOS® FL Color Imaging System; Life Technologies, Carlsbad, CA, USA) or a Zeiss LSM710 confocal microscope system (Carl Zeiss, Munich, Germany). For quantification, two representative areas per slide were randomly selected for five animals and quantified using Image Pro 5.0 software (Media-Cybernetics, Rockville, MD, USA).

Gene expression analyses

Total RNA was extracted using an RNeasy Mini Kit (Qiagen Inc., Valencia, CA, USA) and then reverse transcription was performed using TaqMan Reverse Transcription Reagents (Applied Biosystems, Foster City, CA, USA). RQ-PCR was performed as previously described[11,12]. Relative expression levels of target genes were determined using the $2^{-\Delta\Delta Ct}$ method. GAPDH was used as the endogenous control gene. All primers used in the qPCR assay are listed in the **Table S5**.

Optical imaging and μ-PET/MRI imaging analysis

For bio-imaging experiments, diabetic DUA was induced in 10-week-old female BALB/c-nu (CAnN.Cg-Foxn1nu/CrlCrlj) immunocompromised mice (Charles River Laboratories, Yokohama, Japan). Subsequently, 1×10^5 hUC-MSCs expressing the Nano-lantern construct were administered for IVIS bioluminescence imaging analysis by directly injecting them into the bladder serosa following the protocol established in the rat model. Bioluminescence resonance energy transfer imaging was conducted using the IVIS Spectrum Preclinical In Vivo Imaging System and Living Imaging 4.4 software (PerkinElmer, Waltham, MA, USA), in accordance with the manufacturer's instructions and a previously published protocol[8].

For animal μ-PET/MRI imaging, 14-week-old female NOD/ShiLtJ-*Prkdc*^{em1AMC}*Il2rg*^{em1AMC} (NSGA) mice, purchased from JA BIO (Suwon-si, Gyeonggi-do, Republic of Korea) were randomly divided into three groups and injected with 1×10^5 naïve or PFO procedure hUC-MSCs or PBS vehicle (n = 5). At 3, 6, and 9 months after injection, μ-MRI/PET imaging was performed using the nanoScanPET/MRI imaging system (1T, MEDISO, Budapest Hungary), as previously described[8]. PET images were reconstructed using Tera-Tomo 3D in full detector mode with all the corrections on high regularization and 8 iterations.

Purification of engrafted hUC-MSCs by the two-stage MACS-FACS procedure

To analyze the single-cell transcriptomes of engrafted MSCs, hUC-MSCs stably expressing GFP were labeled with Resovist, a magnetic nanoparticle, following established protocols[4]. Seven days after transplanting GFP^+/$Resovist^+$ hUC-MSCs, bladders were isolated from three STZ-DUA rats, minced into small pieces, and exposed to 2 mL of accutase (A1110501; Gibco, Waltham, MA, USA) at room temperature for 10 minutes. Erythrocytes were removed using

RBC Lysis Solution (158902; QIAGEN, Hilden, Germany). The resulting single-cell suspensions were centrifuged at 6000 × g for 20 minutes at 4°C and cells were resuspended in 1 mL of MACS buffer (low-glucose DMEM containing 2.5% FBS and 1% HEPES). MACS was executed by placing resuspended cells in a magnetic device (12321D, Invitrogen) at room temperature for 3 minutes, followed by two washes with MACS buffer to eliminate unbound cells. Resovist$^+$ cells were further purified based on expression of GFP by FACS using an ARIAIII Flow Cytometer System (BD Biosciences, San Jose, CA, USA), as previously detailed[4]. Contamination by host cells was assessed by quantifying the expression levels of the rat *B2mg* and human *GAPDH* housekeeping genes via RQ-PCR in purified cell samples after each sorting procedure.

Single-cell transcriptome analysis

Single-cell cDNA libraries were generated using a previously established method[4]. In brief, utilizing the ARIAIII Flow Cytometer System, Resovist$^+$/GFP$^+$ engrafted cells (sorted by MACS-FACS) or trypsinized cultured hUC-MSCs (control) were individually dispensed into a well of a 96-well plate (Thermo Scientific, Waltham, MA, USA) containing 4.5 µL of lysis buffer per well. RNA from each well was amplified into T7-primed single-cell cDNA libraries. Initial screening of Resovist$^+$/GFP$^+$ single-cell cDNA libraries involved assessing expression of rat *B2mg* or genes associated with stem cell function using 20-fold-diluted amplified T7-primed PCR products. Only cDNA libraries with minimal host cell contamination underwent genome-wide analysis. T7-primed libraries eluted after gel electrophoresis were biotin-labeled using a GeneChip® 3' *in vitro* transcription kit (Affymetrix, Santa Clara, CA, USA), starting from the protocol step titled "*In vitro Transcription to Synthesize Labeled aRNA*".

Biotin-labeled aRNA from single-cell libraries was fragmented and hybridized to the Affymetrix GeneChip® Human Genome U133 Plus 2.0 Array. Microarray image data were processed using a GeneChip GCS3000 Scanner and Command Console software (Affymetrix). Raw data were automatically extracted following the Affymetrix data extraction protocol provided by Affymetrix GeneChip® Command Console® software.

Transcriptome functional analysis and core analyses of gene networks, biofunctions, and canonical pathways were conducted employing MetaCore (Clarivate Analytics, Philadelphia, PA, USA) or GSEA (Broad Institute, Cambridge, MA, USA) microarray software with default settings. In MetaCore analysis, a cut-off value of 1.5-fold upregulation or downregulation and a significance threshold of $p<0.05$ were applied to identify significant genes. GSEA utilized gene sets derived from previously published literature or filtered from a curated functional gene set (C2) database.

For DEGs in each dataset, volcano plots and heatmaps were generated and Mfuzz clustering was performed using the SRPLOT online tool (http://www.bioinformatics.com.cn/srplot) with a cut-off value of $|log2(FC)| > 1.5$, 2, or 4 and $p<0.05$ considered statistically significant. **Data S1** provides details about significant genes and their associated biological processes and pathways from MetaCore analysis, while **Tables S1–S3** contain gene sets analyzed by GSEA.

Statistical analysis

Data are presented as the mean ± standard error of the mean (SEM). Statistical analysis was performed utilizing GraphPad Prism 7.0 software (GraphPad Software, La Jolla, CA, USA) with the non-parametric Mann-Whitney test or a one-way or two-way ANOVA, followed by the Bonferroni post-hoc test. A p-value <0.05 was deemed indicative of statistical significance.

The exact p-values and numbers for the quantification assays are specified in **Data S1**, provided as a distinct Excel file.

Data and code availability

The transcriptome data presented in this investigation have been submitted to the NCBI Gene Expression Omnibus and can be accessed via GEO Series accession number GSE263324. Comprehensive details about the gene sets utilized for GSEA are provided in **Tables S1–S3**. A detailed list of GO categories and the corresponding genes for functional analyses of transcriptome datasets, encompassing gene networks, biofunctions, and canonical pathways, is presented in **Data S1**, provided as a distinct Excel file. Any additional information necessary for reanalysis of the data disclosed in this research paper is obtainable from the Lead Contact upon request.

SUPPLEMENTARY NOTES

Comprehensive understanding of the key pathological mechanisms of a target disease can provide strong evidence for selecting optimal stem cells capable of repairing pathological insults, ensuring sustained therapeutic efficacy, and maintaining safety, which are crucial to successfully translate stem cell therapies into clinical research. Furthermore, in-depth analysis of the *in vivo* behavior and phenotype of stem cells engrafted into injured target tissues is required to elucidate underlying therapeutic mechanisms, tumorigenic risk, and optimal transplantation protocols. In this study, we examined transcriptome changes in diabetic DUA following M-MSC treatment and demonstrated that disturbance in the response to external stimulus or redox homeostasis related to oxidative injury could be a significant pathological mechanism. hUC-derived PFO-MSCs with high GSH dynamics were proven to be an optimal stem cell source for relieving the pathogenic insults of diabetic DUA. Due to their enhanced *in vivo* retention/engraftment capacity, PFO-MSCs exerted pleiotropic effects by directly contributing to bladder muscle regeneration via differentiating into MET-expressing myogenic cells or PD-L1-expressing cells with immunoregulatory activity, as revealed by single-cell transcriptome analysis of engrafted cells.

The pathophysiology of diabetic bladder dysfunction is multifactorial; comprehensive damage of the urothelium, the detrusor, and nerves results in progressive deterioration of bladder function. It is suggested that diabetic bladder dysfunction occurs via a two-stage process encompassing an early compensated state with predominantly storage symptoms with detrusor hypercontractility and a late decompensated stage with predominantly voiding symptoms. Detrusor muscle contraction is altered by persistent oxidative stress, inflammation, and neuropathy, which leads to changes in the structure and function of the diabetic bladder

and results in this decompensated state[13]. Our findings are consistent with the hypotheses that the alternated response to oxidative stress, inflammation, and immunity could result in diabetic DUA and that reversal or alleviation of these changes could regenerate the detrusor and improve its contractility.

Oxidative stress is assumed to play an essential role in the pathogenesis of diabetic cystopathy because persistent hyperglycemia can trigger excessive ROS production via four possible mechanisms: upregulation of the polyol pathway, formation of advanced glycation products, protein kinase C activation, and MAPK-induced nuclear factor erythroid 2-related factor 2 (NRF2) inhibition[14]. Consistently, the transcriptome profile of bladders of STZ-DUA rats exhibited notable changes in NRF2-related functions, including significant upregulation of *Nqo1*, a primary target of NRF2, as well as modified expression of genes governing GSH synthesis and utilization, which are regulated by NRF2. Notably, treatment with MSCs restored the altered expression of genes involved in the NRF2 regulation of oxidative stress response, indicating that oxidative stress plays a crucial role in the pathogenesis of diabetic bladder and is a principal therapeutic target of MSCs.

Currently established treatments for diabetic lower urinary tract dysfunction are oral pharmacotherapy to enable spontaneous voiding by reducing bladder outlet resistance and enhancing detrusor contractility, de-obstruction surgery, and mechanically draining urine via intermittent catheterization or an indwelling catheter[15]. None of the available therapeutic modalities definitely cure diabetic cystopathy; therefore, experimental attempts, such as stem cell therapy and low-intensity extracorporeal shockwave therapy, to overcome this unmet medical need are on-going. In the field of stem cell therapy, participants of clinical trials have been injected with autologous MSCs originating from muscle or adipose tissue[16-18]. However, previous trials mainly focused on idiopathic DUA not diabetic DUA, and only the therapeutic

potential of autologous stem cell injection has been demonstrated without a suggested mode of action. Considering the prevalence of diabetes in the DUA population, thorough investigations of diabetic DUA and its pathophysiology are important.

Previously, we reported the therapeutic potency of hESC-derived M-MSCs for diabetic DUA in a preclinical study[19]. The advantages of ESCs as an alternative source of MSCs are that they can be indefinitely maintained due to their self-renewal capacity and can differentiate into any cell type due to their pluripotency. Recently, clinical trials using hESC derivatives for disorders in several fields including ophthalmology[20,21] and cardiology[22] have been reported. In urology, we reported the first clinical application of transurethral hESC-derived M-MSC injection in three patients with IC/BPS, proving the potential therapeutic efficacy of these cells without any adverse outcomes[9]. Despite these advantages, safety concerns such as the risk of tumorigenesis remain a major obstacle to the therapeutic application of hESC-derived cell therapy[23,24]. This must be thoroughly investigated prior to clinical application.

When assessing the clinical relevance of novel therapies, including stem cell therapy, it is crucial to prioritize safety alongside effectiveness. In stem cell therapy, the therapeutic impact and risk of carcinogenesis are directly linked to the quantity of stem cells employed. Additionally, the time and cost involved in isolating and expanding stem cells increase with higher target dosages. Consequently, determining the optimal concentration that maximizes therapeutic benefits without adverse effects or tumorigenicity, while keeping costs reasonable, is of paramount importance. In a recent study, we demonstrated the synergistic effect of M-MSCs and NAC in a lipopolysaccharide-induced IC/BPS model, allowing a 4-fold reduction in the stem cell dosage without compromising therapeutic efficacy compared with previously optimized dosages[10]. Similarly, in this study, NAC and M-MSCs had a synergistic effect in a STZ-DUA rat model. Concurrent NAC treatment not only improved voiding function and

histology compared with M-MSC monotherapy but also addressed the significant role of oxidative injury, potentially providing pleiotropic outcomes for alleviating the pathogenic progress of diabetic DUA and facilitating regeneration through MSC therapy. Therefore, clinical application of a combination of NAC and MSC could be considered, although the administration route and dosage of NAC for treatment of bladder dysfunction have not been established.

To ensure safety, an additional crucial approach involves utilization of MSCs derived from adult tissues, which exhibit a robust safety record in numerous clinical investigations[25]. Despite their numerous advantages, such as tissue regenerative capabilities and anti-inflammatory and immunomodulatory properties, adult tissue-derived MSCs have certain limitations, notably a decline in functionality during *ex vivo* expansion, which is required to obtain a sufficient number of cells for therapeutic purposes. To overcome this issue, we have described several *ex vivo* expansion methods to preserve the primitiveness of MSCs, including i) enriching and preserving small cells[26], ii) enhancing the antioxidant capacity by performing real-time monitoring of GSH dynamics[3,7], and iii) enhancing cell migration and engraftment activity by priming cells with small molecules[27]. By combining these benefits without performing genetic manipulation, we reported an optimal culture environment for enrichment and expansion of small primitive MSCs with a high antioxidant capacity, termed the PFO procedure[1]. PFO-MSCs exhibit enhanced stemness and immunomodulatory effects for treating allogeneic conflicts, as demonstrated using cell culture-based assays and a humanized GVHD mouse model. In addition, the superior *in vivo* engraftment of PFO-MSCs may be responsible for their improved therapeutic potency in allergic asthma[2].

Consistent with these studies, the current investigation affirmed that the robust antioxidant capability of PFO-MSCs enhances their viability in the presence of oxidative injury.

This enhancement augments the *in vivo* engraftment capacity of these cells, leading to improved therapeutic efficacy. The remarkable *in vivo* engraftment proficiency of PFO-MSCs allows sustained therapeutic effects lasting 2–4 weeks after a single administration. Notably, through standard histological immunostaining assays, PFO-MSCs demonstrated a propensity to directly differentiate into myocytes, a phenomenon that was challenging to confirm in prior studies employing adult tissue-derived MSC therapies. To substantiate this finding at the molecular level, we employed a high-purity isolation and single-cell transcriptome analysis strategy for administered cells engrafted in the bladder, as detailed in our previous research[4]. PFO-MSCs that integrated into animals with diabetic DUA exhibited diminished expression of apoptosis-related genes and concurrently elevated expression of factors crucial for muscle tissue development.

Specifically, MET, a marker of regenerating myocytes, was highly expressed in $hB2M^+$ cells integrated within muscle bundles of animals with diabetic DUA. This finding is line with the previous report that the HGF–MET signaling cascade plays a crucial role in muscle regeneration[28]. HGF/MET signaling plays a pivotal role in myogenic progenitor migration during embryogenesis and the activation of muscle stem cells (MuSCs) following injury[28]. HGF stimulates quiescent skeletal muscle satellite cells, which are resident MuSCs located at the periphery of the myofibers[29]. Following injury, the regulation of HGF proteolytic processing by a systemic protease HGF activator (HGFA) induces an "alerted" state (Galert) in skeletal MuSCs and fibro-adipogenic progenitors (FAPs), which are MSCs in the muscle interstitium[30]. HGF/MET signaling also cooperates with CXCL12/CXCR4 to protect skeletal MuSCs from inflammation-induced damage during regeneration[31]. Additionally, HGF/MET signaling has diverse tissue-specific roles in regulating the neuromuscular system and interacting with the immune system. Several studies have highlighted the influence of

extracellular components like collagen, diffusible cytokines, and growth factors released by neighboring cells, including resident or infiltrating macrophages and FAPs, on MuSC activity[32,33]. Notably, HGF has been shown to promote macrophage transition to the M2 phenotype during adult muscle regeneration[34]. In this regard, understanding HGF/MET signaling in muscle regeneration requires studying both cell-intrinsic mechanisms, such as its interaction with other signaling pathways in MuSCs, and cell-extrinsic mechanisms, including HGF activation, cell sources, and its interaction with the neural and immune systems. Therefore, further investigations are imperative not only to validate the significance of MET expression in PFO-MSC therapy for diabetic DUA but also to refine the PFO procedure for diabetic DUA by elucidating the pathway governing the HGF–MET signaling cascade and by performing treatment with a related small molecule(s) before transplantation of PFO-MSCs.

Another noteworthy molecular signature of engrafted cells in diabetic DUA was upregulation of genes related to immunomodulation and distinct macrophage subtypes. MSCs play a pivotal role in restraining the activation, proliferation, and function of various immune cells, encompassing T-cells, B-cells, natural killer cells, and antigen-presenting cells[35]. The immunosuppressive effects of MSCs involve cell contact-dependent mechanisms mediated by B7-H1 and PD-L1, in addition to secretion of soluble factors such as interleukin-10, transforming growth factor-β, nitric oxide, prostaglandin E2, and indoleamine 2,3-dioxygenase[1,26]. In this study, M1 macrophage infiltration was increased in diabetic DUA. By contrast, PFO-MSC treatment induced M2 macrophage infiltration, underscoring the significance of macrophage subtype reprogramming as a pivotal therapeutic mechanism. Furthermore, engrafted hB2M$^+$ cells co-expressed PD-L1, a well-known factor directly implicated in the beneficial therapeutic effects of MSCs in challenging immune conditions such as asthma and GVHD[1,2]. Notably, these h2BM$^+$/PD-L1$^+$ cells were present in both muscle

bundles and the muscle periphery. Further investigations elucidating the key mechanisms governing PD-L1 expression in PFO-MSCs are imperative to develop novel and safe platforms for enhancing the immunomodulatory functions of these cells.

LIMITATIONS OF THIS STUDY

MSCs are one of the most extensively investigated and applied types of adult stem cells in the field of regenerative medicine. To ensure successful clinical applications, it is imperative to provide robust evidence for selecting the optimal source and developing methodologies encompassing isolation, propagation, maintenance, up-scale production, and functional characterization and qualification of MSCs[36]. Additionally, a sophisticated design for preclinical studies is necessary to make informed decisions regarding the key administration factors such as delivery routes for target organs, dosage, dose frequency, interval, and suspension method[25].

In this regard, the present study has certain limitations. First, there is a lack of data directly comparing the efficacies of hESC-derived M-MSCs and hUC-derived PFO-MSCs, which have been utilized in previous studies. Second, insufficient evidence is provided to validate the synergistic effect of combination therapy with PFO-MSCs and NAC, as well as to optimize the cell quantity for ensuring the efficacy and safety of PFO-MSC therapy. Third, considering that the PFO procedure is applicable to MSCs sourced from various adult tissues, there is a lack of results concerning the optimal selection of adult tissues for obtaining PFO-MSCs to specifically treat diabetic DUA. Finally, there is a scarcity of conclusive experimental findings regarding the synchronization of cells integrated into muscle bundles with the host muscular network and their direct contribution to functional regeneration. All these data are

indispensable for clinical studies utilizing PFO-MSCs to treat diabetic DUA. Further research efforts are required to acquire and leverage this information in order to develop a standard operating procedure suitable for producing good manufacturing practice-grade PFO-MSCs.

SUPPLEMENTARY REFERENCES

1. Lim J, Heo J, Yu HY, et al. Small-sized mesenchymal stem cells with high glutathione dynamics show improved therapeutic potency in graft-versus-host disease. *Clin Transl Med*. 2021;11:e476.
2. Ju H, Yun H, Kim Y, et al. Activating transcription factor-2 supports the antioxidant capacity and ability of human mesenchymal stem cells to prevent asthmatic airway inflammation. *Exp Mol Med*. 2023;55:413-425.
3. Jeong EM, Yoon JH, Lim J, et al. Real-Time Monitoring of Glutathione in Living Cells Reveals that High Glutathione Levels Are Required to Maintain Stem Cell Function. *Stem Cell Reports*. 2018;10:600-614.
4. Yu HY, Lee S, Ju H, et al. Intravital imaging and single cell transcriptomic analysis for engraftment of mesenchymal stem cells in an animal model of interstitial cystitis/bladder pain syndrome. *Biomaterials*. 2022;280:121277.
5. Shin DM, Liu R, Wu W, et al. Global gene expression analysis of very small embryonic-like stem cells reveals that the Ezh2-dependent bivalent domain mechanism contributes to their pluripotent state. *Stem Cells Dev*. 2012;21:1639-52.
6. Lee S, Lim J, Lee JH, et al. Ascorbic Acid 2-Glucoside Stably Promotes the Primitiveness of Embryonic and Mesenchymal Stem Cells Through Ten-Eleven Translocation- and cAMP-Responsive Element-Binding Protein-1-Dependent Mechanisms. *Antioxid Redox Signal*. 2020;32:35-59.
7. Lim J, Heo J, Ju H, et al. Glutathione dynamics determine the therapeutic efficacy of mesenchymal stem cells for graft-versus-host disease via CREB1-NRF2 pathway. *Science Advances*. 2020;6:eaba1334.

8. Kim A, Yu HY, Lim J, et al. Improved efficacy and in vivo cellular properties of human embryonic stem cell derivative in a preclinical model of bladder pain syndrome. *Sci Rep*. 2017;7:8872.
9. Shin JH, Ryu CM, Yu HY, et al. Safety of Human Embryonic Stem Cell-derived Mesenchymal Stem Cells for Treating Interstitial Cystitis: A Phase I Study. *Stem Cells Transl Med*. 2022;11:1010-1020.
10. Shin JH, Ryu CM, Ju H, et al. Synergistic Effects of N-Acetylcysteine and Mesenchymal Stem Cell in a Lipopolysaccharide-Induced Interstitial Cystitis Rat Model. *Cells*. 2019;9:86.
11. Heo J, Lee J, Nam YJ, et al. The CDK1/TFCP2L1/ID2 cascade offers a novel combination therapy strategy in a preclinical model of bladder cancer. *Exp Mol Med*. 2022;54:801-811.
12. Kim Y, Ju H, Yoo SY, et al. Glutathione dynamics is a potential predictive and therapeutic trait for neoadjuvant chemotherapy response in bladder cancer. *Cell Rep Med*. 2023;4:101224.
13. Klee NS, McCarthy CG, Lewis S, McKenzie JL, Vincent JE, Webb RC. Urothelial Senescence in the Pathophysiology of Diabetic Bladder Dysfunction-A Novel Hypothesis. *Front Surg*. 2018;5:72.
14. Song QX, Sun Y, Deng K, Mei JY, Chermansky CJ, Damaser MS. Potential role of oxidative stress in the pathogenesis of diabetic bladder dysfunction. *Nat Rev Urol*. 2022;19:581-596.
15. Erdogan BR, Liu G, Arioglu-Inan E, Michel MC. Established and emerging treatments for diabetes-associated lower urinary tract dysfunction. *Naunyn Schmiedebergs Arch Pharmacol*. 2022;395:887-906.
16. Coelho HRS, Neves SCD, da Silva Menezes JN, Antoniolli-Silva A, Oliveira RJ. Autologous adipose-derived mesenchymal stem cell therapy reverses detrusor underactivity: open clinical trial. *Stem Cell Res Ther*. 2023;14:64.
17. Gilleran J, Diokno AC, Ward E, et al. Improved global response outcome after intradetrusor injection of adult muscle-derived cells for the treatment of underactive bladder. *Int Urol Nephrol*. 2021;53:1331-1338.
18. Levanovich PE, Diokno A, Hasenau DL, Lajiness M, Pruchnic R, Chancellor MB. Intradetrusor injection of adult muscle-derived cells for the treatment of underactive

bladder: pilot study. *Int Urol Nephrol*. 2015;47:465-7.
19. Shin JH, Ryu CM, Ju H, et al. Therapeutic Efficacy of Human Embryonic Stem Cell-Derived Multipotent Stem/Stromal Cells in Diabetic Detrusor Underactivity: A Preclinical Study. *Journal of clinical medicine*. 2020;9.
20. Schwartz SD, Regillo CD, Lam BL, et al. Human embryonic stem cell-derived retinal pigment epithelium in patients with age-related macular degeneration and Stargardt's macular dystrophy: follow-up of two open-label phase 1/2 studies. *Lancet*. 2015;385:509-16.
21. Schwartz SD, Hubschman JP, Heilwell G, et al. Embryonic stem cell trials for macular degeneration: a preliminary report. *Lancet*. 2012;379:713-20.
22. Menasche P, Vanneaux V, Hagege A, et al. Human embryonic stem cell-derived cardiac progenitors for severe heart failure treatment: first clinical case report. *Eur Heart J*. 2015;36:2011-7.
23. Desgres M, Menasche P. Clinical Translation of Pluripotent Stem Cell Therapies: Challenges and Considerations. *Cell Stem Cell*. 2019;25:594-606.
24. Heo J, Lim J, Lee S, et al. Sirt1 Regulates DNA Methylation and Differentiation Potential of Embryonic Stem Cells by Antagonizing Dnmt3l. *Cell Rep*. 2017;18:1930-1945.
25. Han S, Yim HW, Jeong H, Choi S, Han S. Establishing Rationale for the Clinical Development of Cell Therapy Products: Consensus between Risk and Benefit. *Int J Stem Cells*. 2023;16:16-26.
26. Kim Y, Jin HJ, Heo J, et al. Small hypoxia-primed mesenchymal stem cells attenuate graft-versus-host disease. *Leukemia*. 2018;32:2672-2684.
27. Lim J, Lee S, Ju H, et al. Valproic acid enforces the priming effect of sphingosine-1 phosphate on human mesenchymal stem cells. *Int J Mol Med*. 2017;40:739-747.
28. Relaix F, Bencze M, Borok MJ, et al. Perspectives on skeletal muscle stem cells. *Nat Commun*. 2021;12:692.
29. Birchmeier C, Birchmeier W, Gherardi E, Vande Woude GF. Met, metastasis, motility and more. *Nat Rev Mol Cell Biol*. 2003;4:915-25.
30. Rodgers JT, Schroeder MD, Ma C, Rando TA. HGFA Is an Injury-Regulated Systemic Factor that Induces the Transition of Stem Cells into G(Alert). *Cell Rep*. 2017;19:479-486.

31. Lahmann I, Griger J, Chen JS, Zhang Y, Schuelke M, Birchmeier C. Met and Cxcr4 cooperate to protect skeletal muscle stem cells against inflammation-induced damage during regeneration. *Elife*. 2021;10.
32. Kim E, Wu F, Lim D, et al. Fibroadipogenic Progenitors Regulate the Basal Proliferation of Satellite Cells and Homeostasis of Pharyngeal Muscles via HGF Secretion. *Front Cell Dev Biol*. 2022;10:875209.
33. Sefton EM, Gallardo M, Tobin CE, et al. Fibroblast-derived Hgf controls recruitment and expansion of muscle during morphogenesis of the mammalian diaphragm. *Elife*. 2022;11.
34. Choi W, Lee J, Lee J, Lee SH, Kim S. Hepatocyte Growth Factor Regulates Macrophage Transition to the M2 Phenotype and Promotes Murine Skeletal Muscle Regeneration. *Front Physiol*. 2019;10:914.
35. Kim OH, Jeon TJ, So YI, Shin YK, Lee HJ. Applications of Bioinspired Platforms for Enhancing Immunomodulatory Function of Mesenchymal Stromal Cells. *Int J Stem Cells*. 2023;16:251-259.
36. Jovic D, Yu Y, Wang D, et al. A Brief Overview of Global Trends in MSC-Based Cell Therapy. *Stem Cell Rev Rep*. 2022;18:1525-1545.
